# Isolation of Methane from Ambient Water and Preparation for Source-Diagnostic Natural Abundance Radiocarbon Analysis

**DOI:** 10.1021/acs.analchem.4c03525

**Published:** 2024-10-24

**Authors:** Marenka Brussee, Henry Holmstrand, Michael Süß, Amelia Davies, Örjan Gustafsson

**Affiliations:** † Department of Environmental Science, Stockholm University, Stockholm 10691, Sweden; ‡ Bolin Centre for Climate Research, Stockholm University, Stockholm 10691, Sweden

## Abstract

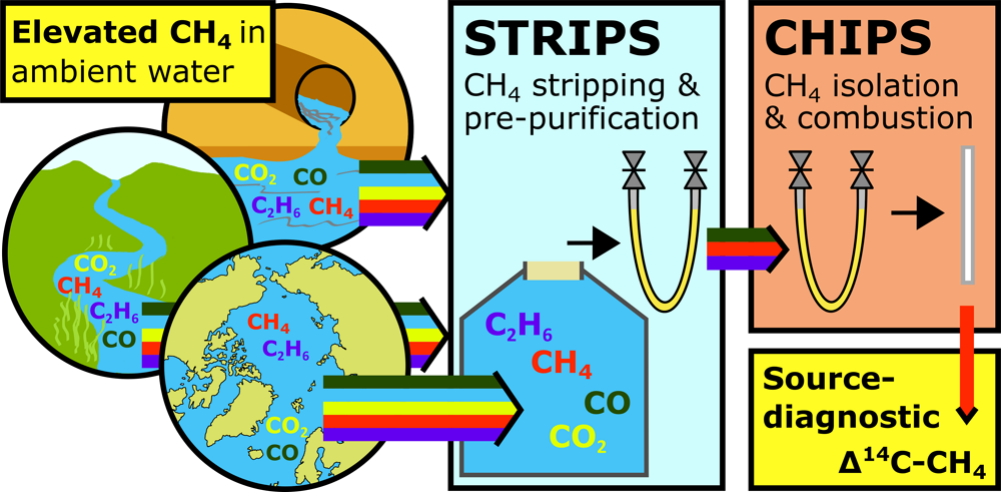

A key challenge in climate change research is apportioning the greenhouse gas methane (CH_4_) between various natural and anthropogenic sources. Isotopic source fingerprinting of CH_4_ releases, particularly with radiocarbon analysis, is a promising approach. Here, we establish an analytical protocol for preparing CH_4_ from seawater and other aqueous matrices for high-precision natural abundance radiocarbon measurement. Methane is stripped from water in the optionally field-operated system (STRIPS), followed by shore-based purification and conversion to carbon dioxide (CO_2_) in the CH_4_ Isotope Preparation System (CHIPS) to allow Accelerator Mass Spectrometry analysis. The blank (±1σ) of the combined STRIPS and CHIPS is low (0.67 ± 0.12 μg C), allowing natural sample sizes down to 10 μg C–CH_4_ (i.e., 30 L samples of 40 nM CH_4_). The full-system yield is >90% for both CH_4_-spiked seawater and ambient samples from CH_4_ hotspots in the Baltic Sea and the Arctic Ocean. Furthermore, the radiocarbon isotope signal of CH_4_ remains constant through the multistage processing in the STRIPS and the CHIPS. The developed method thus allows for in-field sampling and sample size reduction followed by precise and CH_4_-specific radiocarbon analysis. This enables powerful source apportionment of CH_4_ emitted from aquatic systems from the tropics to the polar regions.

## Introduction

The global atmospheric budget of the strong greenhouse gas methane (CH_4_) has large uncertainties.^1,2^ Moreover, global atmospheric CH_4_ concentrations have, since 2006, shown an accelerated increase coincident with a negative shift in its stable carbon isotope (δ^13^C) composition.^2−4^ This likely reflects enhanced CH_4_ releases from microbial sources from either anthropogenic (e.g., rice paddies, cattle) and/or natural (e.g., tropical-boreal wetlands/lakes, thawing land/subsea permafrost) systems, possibly triggered by climate warming. Compound-specific radiocarbon analysis (CSRA) of CH_4_ may be used to apportion contributions of different sources^5^ and is effective as Δ^14^C measurements are corrected for isotopic fractionation and offer age constraints.^5,6^

The here described system for CSRA of CH_4_, while broadly applicable to many kinds of aquatic systems (Supporting Information (SI) Table S14), was primarily developed for application in the Arctic, warming three times faster than the global average,^7^ possibly accelerating CH_4_ release in areas with permafrost. It is particularly poorly understood how and to which extent CH_4_ is released from the extensive Arctic shallow seas underlain by subsea permafrost, formed 14–8 ky BP.^8^ Estimates of CH_4_ fluxes to the atmosphere for these shallow seas currently span a wide range (5 × 10^–2^ to 3 × 10^2^ Tg CH_4_/y)^9^ and may thus be significant in the context of the global estimates of the net atmospheric CH_4_ increase (10^1^–10^2^ Tg CH_4_/y).^1,2^ Elevated CH_4_ levels across extensive Arctic shelf seas and in thermokarst lakes around the circum-Arctic may stem from young sources (such as in situ aerobic methanogenesis or anaerobic methanogenesis in sediments) or intermediate old to ancient sources (from thawed permafrost, CH_4_ hydrates or natural gas leakage).^10−13^ So far, the few pioneering studies using CSRA of dissolved CH_4_ in Arctic coastal waters^12,13^ have been inconclusive regarding the dominant sources of CH_4_ in the circum-Arctic shelf seas, highlighting the need to develop and apply CSRA of aqueous CH_4_.

Existing methods to isolate CH_4_ from natural water systems and quantitatively convert it to carbon dioxide (CO_2_) for CSRA face analytical challenges such as long sampling times,^14^ lack of demonstrated removal of carbon monoxide (CO)^15,16^ and nonmethane hydrocarbons (NMHCs)^15^ coexisting in natural waters, and high or even absent reporting of blank levels of the total procedures^13−17^ (SI Table S13). Total process blanks (±1σ) reported in earlier studies range from 6.3 ± 4.7^17^ and 5.0 ± 2.4^14^ μg C to no reports of complete process blanks.^13,15,16^ Such uncertainties and blank levels limit the accuracy, precision, and reliability of existing Δ^14^C–CH_4_ methods and would, at the very least, require substantial sample sizes to overcome the process blank contamination (of often unknown Δ^14^C). This complicates sampling in often hard-to-access field conditions and during marine expeditions, where it is desired to sample seawater quickly to make the most of the ship time and generate data sets with good geographic resolution. An improved analytical ability to effectively apply CSRA to fresh- and seawater CH_4_ would facilitate the generation of robust isotopic data sets which enhance our understanding of the contributing sources to observed CH_4_ releases in the Arctic shelf seas^13,18^ and elsewhere, thereby facilitating the anticipation of future CH_4_ release trajectories and potential carbon-climate feedbacks.

The here-developed preparation system for CSRA of dissolved CH_4_ in ambient water is optimized for rapid sampling, effective sample gas extraction, and in-field sample reduction while preserving the highest possible CSRA resolving power down to C–CH_4_ sample amounts as low as 10 μg C, which is the minimum sample size required for radiocarbon analysis with Accelerator Mass Spectrometry (AMS). The system exists of two parts. The subsystem STRIPS is a headspace extraction system for CH_4_ extraction from 30 L ambient water samples and includes prepurification of CH_4_ by bulk removal of water (H_2_O) vapor and CO_2_. The STRIPS part is a modification of earlier designs,^13,17^ which is further optimized for a much lower blank contribution and maximum removal of H_2_O vapor and CO_2_ from the headspace gas. The subsequent subsystem CHIPS is a purification system where the in-STRIPS extracted gas is cleaned from residual non-CH_4_ carbon, and purified CH_4_ is quantitatively converted to CO_2_ to prepare the sample for radiocarbon analysis by AMS. Central components are constraining and minimizing the extraneous carbon associated with the sample water and procedure to achieve a consistent total process blank <1 μg C, facilitating more extensive sampling coverage and more accurate CSRA. Besides showing the functioning and quality of various subparts and the whole procedure, we demonstrate the application of the new method to ambient water samples.

## Experimental Section

### Description of the CH_4_ Stripping and Purification System (STRIPS)

**Figure 1:**
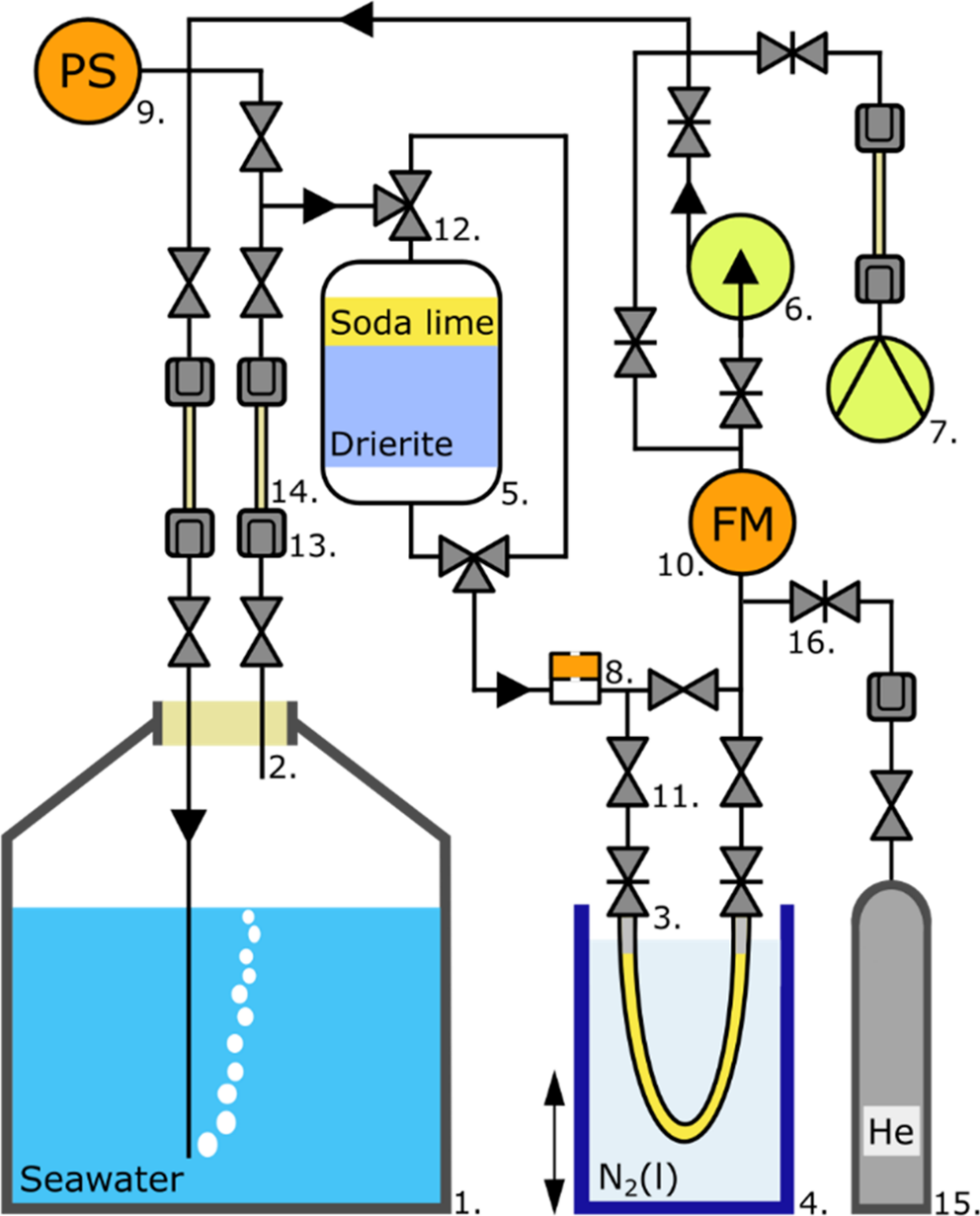
Flowchart of the STRIPS: 1. Keg with seawater and He headspace, 2. Customized keg headpiece, 3. Customized U-trap with two needle valves, 4. LN_2_-filled Dewar flask, 5. Gas purifying canister containing Drierite and soda lime, 6. Circulation pump, 7. Vacuum pump, 8. Septum for sampling with syringe, 9. Pressure sensor (PS, digital), 10. Flowmeter (FM), 11. Toggle valve, 12. 3-way valve, 13. Quick-connect fitting, 14. Perfluoroalkoxy (PFA) tubing, 15. Gas cylinder with He grade 5.0, 16. Needle valve. Note: All single black lines represent 1:4 in. stainless steel tubing, a mixed set of flexible bellows and stiff tubing. Details regarding the construction parts, product names, and suppliers can be found in the Supporting Information (Table S1).

The goal of the optionally field-operated CH_4_ Stripping and Purification System (STRIPS, Figure 1) is to extract CH_4_ from ambient water samples and trap this gas in an easily transportable and storable stainless-steel U-trap (No. 3 in Figure 1). Aqueous samples are collected in modified 30 L stainless-steel kegs (No. 1) and temporarily stored with custom-made lids (SI Table S1). Kegs may be filled with sample water from either Niskin or Go-Flo flasks (SI Table S12). The kegs are robust and thus facilitate field handling and transport if CH_4_ extraction is not carried out on-site.

During the STRIPS operation, the keg has a helium (He) headspace and is fitted with a custom-made headpiece (No. 2 in Figure 1). The headpiece consists of two stainless steel tubes, each with a toggle valve, assembled through the custom-made lid, where the longer tube extends to the bottom of the water inside the keg while the short tube ends just below the lid. The keg headpiece has quick-connect fittings (No. 13), facilitating operation in the field.

The STRIPS is a circular system in which He gas is repeatedly looped through the aqueous sample. The circulation pump (No. 6) and flowmeter (No. 10) control and monitor the flow rate. A septum (No. 8) allows gas sampling for measurements of concentrations of CO_2_, CO, CH_4_, and ethane (C_2_H_6_) with a Gas Chromatography Flame Ionization Detector (GC-FID). A pressure transducer and datalogger (No. 9) allow pressure monitoring. These measurements, combined with trapping times in the U-trap, enable estimation of the amount of CO_2_, CO, CH_4_, and C_2_H_6_ trapped in the U-trap when operating the STRIPS.

The CH_4_, extracted from the water, is cleaned from bulk H_2_O and CO_2_ by Drierite and soda lime, held in an acrylic gas purifying unit (No. 5), and collected in the U-trap (No. 3). This custom-made U-trap consists of a bent stainless-steel tube filled with zeolite “HiSiv 3000” with needle valves at both ends and is cooled in liquid nitrogen (LN_2_) to adsorb CH_4_ on the zeolite. The U-trap is the intermediate sample container, which is subsequently attached to the CHIPS for further processing.

The STRIPS resembles some earlier headspace extraction systems^13,17^ yet differs both in construction parts (e.g., the use of soda lime and cap, the type of container, headpiece, and quick-connect fittings), as described above, and in operational procedures (e.g., the headspace creation method, the circulation at above ambient pressure, and the 1 h of H_2_O and CO_2_ removal before CH_4_ extraction), as elaborated on in the next section. The individual components and the complete method have been tested for the STRIPS (SI Table S7).

### Operational Protocol for the STRIPS

**Figure 2:**
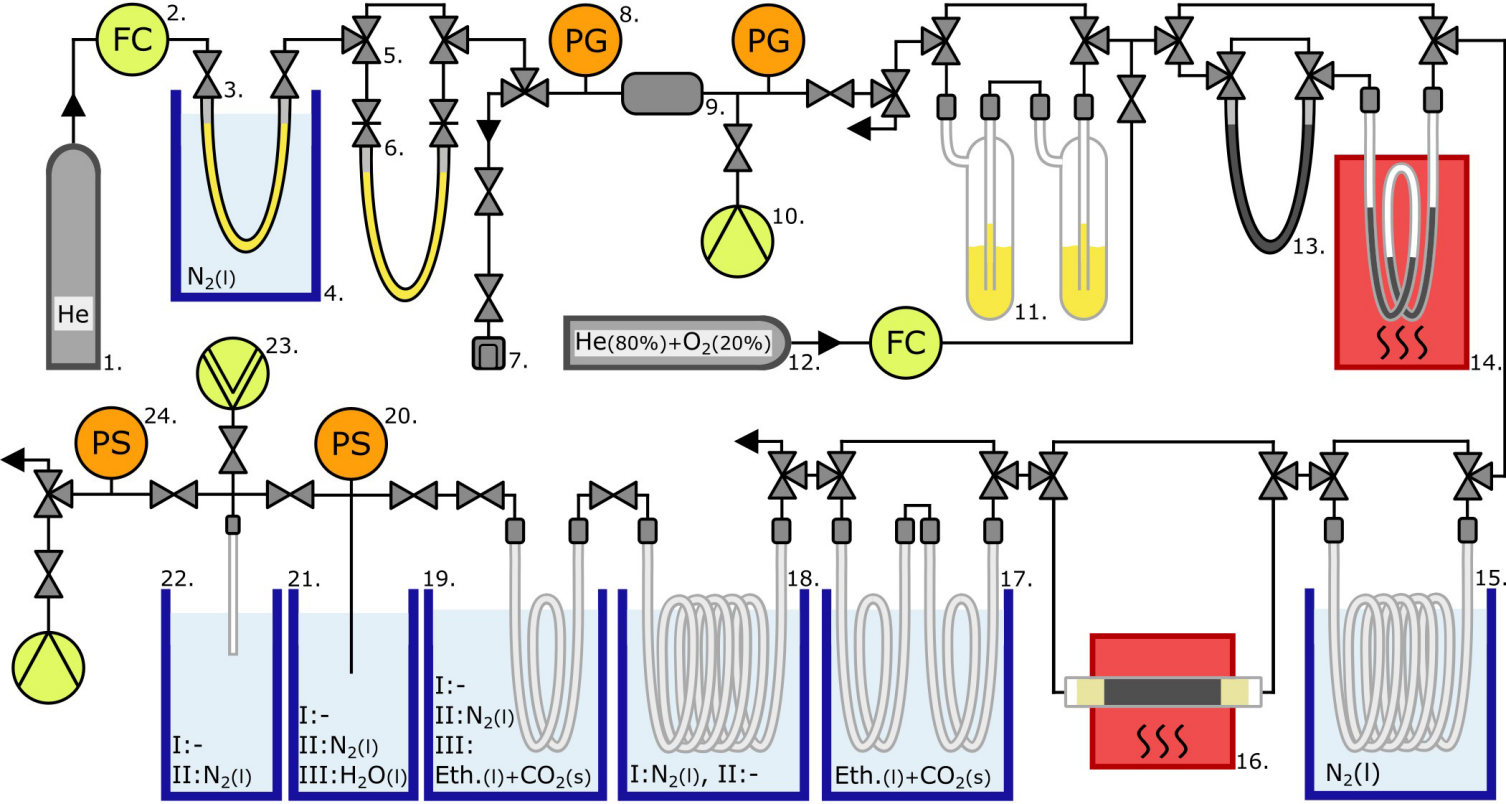
Flowchart of the CHIPS: 1. Gas cylinder with He grade 5.0, 2. Flow controller (FC), 3. Toggle valve, 4. CH_4_-scrubber cooled in LN_2_-filled dewar flask, 5. 3-way valve, 6. Customized U-trap with two needle valves, 7. Quick-connect fitting, 8. Pressure gauge (PG), 9. Expansion volume, 10. Vacuum pump, 11. CO_2_-scrubber, 12. Gas cylinder with 80% He grade 6.0 and 20% O_2_ grade 5.2, 13. CO-removal trap, 14. Mild oven at 430 °C with CuO, 15. LN_2_-cooled CO_2_-scrubber, 16. Main oven at 975 °C with platinized quartz wool, 17. H_2_O-scrubbers, 18. Large LN_2_-cooled CH_4_-derived CO_2_ collector, 19. Small LN_2_-cooled CH_4_-derived CO_2_ collector, 20. Small-range pressure sensor (PS, digital), 21. Manometric assembly, 22. Ampule, 23. High-vacuum pump, 24. High-range pressure sensor (PS, digital). Note: The single black lines represent a mixed set of flexible bellows and stiff 1:4 in. stainless steel tubing and flexible 1:4 in. Synflex tubing. Details regarding the construction parts, product names, and suppliers can be found in the Supporting Information (Table S2).

Several preparation steps are executed before processing a sample in the STRIPS (SI Table S3). A leak-tested and cleaned U-trap (No. 3 in Figure 1) is attached to the STRIPS, and the gas purifying unit (No. 5) is refilled with new and (regenerated) Drierite. The STRIPS is then leak-checked before replacing the keg lid with the customized headpiece (No. 2). In the keg, a ∼9.5 L He headspace is created by purging He through the short tube of the headpiece, forcing sample water to be expelled. The controlled flow of grade 5.0 He is stripped of any trace CH_4_ by leading the He flow through an LN_2_-cooled U-trap. This flow is realized using the CHIPS’s front part (Nos. 1–7 in Figure 2) or, in the field, an isolated headspace creation system (SI Figure S3). This results in a starting pressure in the keg of ∼1.1 bar. After the attachment of the keg to the STRIPS, a second leak test of the STRIPS is executed. The last preparation step is cleaning the STRIPS from contamination of ambient air by the use of He gas (No. 15), circulation pump (No. 6), and vacuum pump (No. 7).

The actual CH_4_ extraction protocol (SI Table S4) then consists of two main phases. First, gases are equilibrated between dissolved and gaseous phases while removing CO_2_ and H_2_O by circulating He gas through the aqueous sample and the gas purifying canister. After 1 h of equilibration between the gas and water phase, the headspace gas is sampled for GC-FID analysis, and the pressure is measured. During the second main phase, the gas flow in the STRIPS is guided through the LN_2_-cooled U-trap for 1 h. Headspace gas is analyzed after the trapping to ensure complete trapping of CH_4_ in the U-trap and to monitor the CO_2_ level in the system. The U-trap is thereafter closed and stored at room temperature until processing in the CHIPS.

### Description of the CH_4_ Isotope Preparation System (CHIPS)

The goal of the CH_4_ Isotope Preparation System (CHIPS, Figure 2) is to purify the STRIPS-isolated sample further, oxidize the purified CH_4_, and collect the CH_4_-derived CO_2_ in a sample format that can be analyzed by AMS. The individual components of the CHIPS are summarized below, with the full details provided in SI Table S2, while the tests of these components are described in SI Table S8.

In contrast to the circular STRIPS system, the CHIPS has a linear design with two flow-controlled gas streams and two exits. The main He stream enters at No. 1 (Figure 2) and a mixture of oxygen (O_2_) and He at No. 12. Incoming He is scrubbed from trace CH_4_ by purging the flow through an LN_2_-cooled U-trap containing zeolite “HiSiv 3000” (No. 4). The sample-holding U-trap from the STRIPS is attached at No. 6. This is upstream of an expansion volume (No. 9) incorporated as a safety measure for release of potentially cotrapped gases.

In multiple stages, gas released from the U-trap is cleaned of nonmethane carbon sources (NMCs) like CO, C_2_H_6_, and CO_2_. There are two borosilicate glass “Russian doll” traps, filled to a third with zeolite “13X APG”, to trap CO_2_ (No. 11). This zeolite is known to be a CO_2_-trapping but CH_4_-conserving molecular sieve which also traps H_2_O.^14^ Thereafter two catalysts for CO and NMHCs removal follow, which can optionally be excluded in case no CO or NMHCs are present in the studied sample. The first catalyst for CO removal is Sofnocat (No. 13), loaded in a stainless steel tube. The second catalyst for removing both CO and NMHCs is CuO (No. 14), heated in a borosilicate glass Vertical Coil (VC)-2 trap to 430 °C in a custom-made oven. It has been shown that Sofnocat is very effective in removing CO^19,20^ and that CuO at 290 °C oxidizes CO and NMHCs to CO_2._^17^ Any remaining NMHCs not oxidized in the mild oven (No. 14) are removed by an LN_2_-cooled^14^ VC-6 trap (No. 15), which also removes CO_2_. After removing extraneous carbon through these steps, the purified CH_4_ is ready for oxidation.

The purified CH_4_ is oxidized to CO_2_ in an oven at 975 °C, containing a quartz glass tube filled with platinized quartz wool as a catalyst (No. 16). Platinized quartz wool is selected because of its high CH_4_ conversion efficiency and known lack of memory effects.^14,20^ The H_2_O formed during oxidation is removed in two consecutive VC-2 traps (No. 17) by cooling in a slurry of ethanol and dry ice^14,16^ at ∼−70 °C.

The CH_4_-derived CO_2_ is cryo-trapped in two stages of the CHIPS. The CH_4_-derived CO_2_ is first trapped in an LN_2_-cooled VC-6 trap (No. 18). The design of this trap (No. 18) is identical to the trap at No. 15 as the purposes are similar; complete trapping of either CH_4_-derived CO_2_ or contamination CO_2_. After that follows a VC-2 trap (No. 19) for secondary collection of CH_4_-derived CO_2_. This trap is only LN_2_-cooled during the subtransfer of CO_2_ from the VC-6 trap (No. 18) to the VC-2 trap (No. 19).

The CH_4_-derived CO_2_ is then collected and quantified in a manometric assembly (Nos. 20–21). After quantification, the CH_4_-derived CO_2_ is flame-sealed in a borosilicate ampule (No. 22), the deliverable for radiocarbon analysis by AMS. Two vacuum pumps are used to evacuate the CHIPS overnight and before any cryo-transfer, manometric quantification, or ampule sealing. A low-vacuum diaphragm pump is used to reduce pressures from above ambient to ∼10 mbar (No. 10), and a high-vacuum combined turbomolecular and diaphragm pump reduces the pressure further to <0.01 mbar (No. 23).

### Operational Protocol for the CHIPS

Several preparation steps are executed before attaching a sample to the CHIPS (SI Table S5). The CHIPS is evacuated overnight and during periods of nonoperation to clean the system and regenerate the adsorbents with pressure swings. The CHIPS is leak-checked, and after that, a small flow of He at low pressure is started while the ovens (Nos. 14 and 16) are turned on to clean the entire line and regenerate the adsorbents further. Then, the ovens are baked out and conditioned under He and O_2_ at their operational temperatures. The last preparation step is precooling the CH_4_- and CO_2_-scrubbers (Nos. 4 and 15).

The entire operational procedure for processing a sample through the CHIPS, including each step’s purpose, is detailed in SI Table S6. For quality control purposes, a series of sample runs with the CHIPS always start with a blank run to assess and ensure a low C signal before processing any sample. After that, up to three samples are processed through the CHIPS. The operational procedure for a blank run is identical to processing a sample through the CHIPS, except that no U-trap (sample from the STRIPS) is attached and opened.

### Novel Features of the CHIPS

There are several new aspects in the design and operational procedure of the CHIPS relative to earlier approaches. In contrast to former methods,^14,20^ which operate at a pressure <300 mbar to omit O_2_ condensation, the CHIPS is operated slightly above ambient pressure (∼1250 mbar at No. 2 in Figure 2). As our O_2_ concentration is ∼3.9%, the partial pressure of O_2_ is at all times <50 mbar, much smaller than the vapor pressure of O_2_ at LN_2_ temperature (∼200 mbar),^21^ preventing condensation of O_2_. Operation at slightly above ambient pressure is favorable as this limits pressure-induced leakage into a system (and thus contamination). Nevertheless, as a safety measure, we always evacuate any LN_2_-cooled component of the CHIPS to <1 mbar before any LN_2_ removal. Working above ambient pressure has likely contributed to the low CHIPS blank (see the Results section).

A second novel aspect in the design of the CHIPS is the two LN_2_-cooled VC-6 traps (Nos. 15 and 18 in Figure 2 and SI Figures S2.3 and S2.4), which are uniquely designed to maximize CO_2_ trapping at ∼2 LPM. A potential disadvantage of the VC-6 trap at No. 18 is a longer cryo-transfer to the manometric assembly. As this step takes place at subambient pressure, longer transfer times bring a risk for increased ambient CO_2_ contamination. The subtransfer from the VC-6 trap (No. 18) to the small VC-2 trap (No. 19) with a small He flow above ambient pressure reduces this risk. Furthermore, the LN_2_ bath around this VC-2 trap (No. 19) is more easily replaced with an ethanol and dry ice slurry to retain potentially cotrapped H_2_O.^14,17^ The VC-6 traps, combined with subtransfer, have likely contributed to a low CHIPS blank and high full-system yield.

A third novel aspect concerns the routing of gas through the CHIPS, which is optimized to minimize the extraneous carbon blank further. The CHIPS method protocol minimizes both the total amount of O_2_ and the time gases pass through the ovens. This is achieved by routing the gas flow through the CHIPS such that the gas (He or combined He and O_2_) flows only through the set of necessary subparts. This necessitates additional valve manipulations and flow rerouting during the sample processing yet helps to achieve a lower and more stable blank.

### Full-System Blanks and Recovery Tests

Five blank samples were prepared from helium-purged seawater (SI Table S9) to test the total carbon blank of the STRIPS and the CHIPS. To assess the combined recovery of CH_4_ of the complete STRIPS and CHIPS analytical sequence, five samples with seawater were spiked with CH_4_ with Δ^14^C–CH_4_ <−997.3‰ (SI Table S9). The ^14^C-free CH_4_ was selected to resolve any ambient air contamination as that would give a more modern ^14^C signal. Furthermore, as shown in the results, the small carbon blank for the CHIPS was also relatively ^14^C modern. For three of the five samples, ∼50 mL ambient air was added after the CH_4_ injection to simulate the maximum ambient air amount that could enter the keg during the lid-to-headpiece change for field-sampled kegs (SI Table S11). Seawater is considered an authentic matrix for both the full-system blanks and recovery tests. Details regarding the U-trap storage time after the STRIPS operation and the included CHIPS components for these full-system tests are shown in SI Tables S10 and S11.

Ambient seawater with high CH_4_ concentrations for method testing was obtained from the anoxic Landsort Deep (58.6°N, 18.2°E) in the central Baltic Sea and oxic bottom water at known CH_4_ hotspots^18^ in the Laptev Sea, Arctic Ocean. For the Baltic Sea, two sample kegs were filled with seawater sampled at 430 and 150 m depth in March 2020. In October 2019, two kegs were filled with water from the Laptev Sea (locations 73.1°N, 130.5°E, and 76.8°N, 125.9°E) sampled at 21 and 66 m depth, respectively. The procedure for preparing and filling the sample kegs is described in SI Table S12. These kegs were analyzed according to the STRIPS and CHIPS operational protocols, and details regarding the storage time of the filled U-traps and catalysts included in the CHIPS are in SI Table S11.

For each yield computation of the total recovery tests, the blank size of the corresponding specific experimental setup, as detailed in SI Table S11, was used in combination with the AMS-determined blank Δ^14^C value of −231 ± 141‰ (SI Table S8). For the CH_4_-spiked samples, four of the five samples were flame-sealed in the CHIPS, and three of these were analyzed by AMS (Table 1). The CH_4_ radiocarbon results for the Baltic Sea and Arctic Ocean samples are in Table 1. All radiocarbon data were generated at the Tandem Laboratory (Faculty of Science and Technology) of Uppsala University.

## Results and Discussion

### Method Tests of the STRIPS

The STRIPS had a negligible blank, a high CH_4_ recovery (>95% yield), and was >99% effective in removing CO_2_ and H_2_O. The effectiveness of the removal was determined by Cavity Ring-Down Spectrometry (CRDS) and was 99.6 ± 0.6% (1σ, *n* = 3) for CO_2_ and 99.2 ± 0.3% (1σ, *n* = 3) for H_2_O (SI Table S7). Furthermore, GC-FID analysis of the STRIPS headspace gas showed that the CO_2_ concentration was always lower than 4 ppm for the combined STRIPS and CHIPS experiments. The maximum amount of CO_2_ that could be trapped in a U-trap and introduced to the CHIPS was estimated to be 0.5 mL pure CO_2_ gas based on the maximum CO_2_ concentration (4 ppm), operational flow rate, and trapping times. As shown below, such residual CO_2_ amounts are not a concern as these are effectively removed in the CHIPS. The addition of soda lime to remove CO_2_ from the gas stream is a significant improvement compared to a former setup where no removal of CO_2_ during the CH_4_ extraction phase was executed^13^ as it reduces the amount of CO_2_ brought forward and thus eases purification of CH_4_ in the CHIPS.

The developed STRIPS, including sample U-trap, was effective in stripping and releasing CH_4_. The trapping and release yield in the STRIPS for the CH_4_-spiked seawater-filled kegs and >4 weeks-storage of U-traps was 96.9 ± 3.0% (1σ, *n* = 3, SI Table S7). Furthermore, the trapping and release yield for He-filled kegs and up to 24 h U-trap storage was 97.7 ± 1.2% (1σ, *n* = 3, SI Table S7). This demonstrates the high yield for trapping CH_4_ in the STRIPS for kegs with seawater-dissolved CH_4_ and long U-trap storage. During the cryo-trapping phase of these experiments, the CH_4_ concentration in the gas stream was reduced by >99.4% (SI Table S7), further supporting the effectiveness of the stripping. A potentially slightly lower yield for the combined trapping and release compared to trapping only could be due to gas leakage out of the STRIPS as the STRIPS is operated at a slight overpressure or incomplete CH_4_ release from the adsorbent in the U-trap. This highlights the need to extensively clean U-traps to reduce potential cross-contamination. Both the trapping of CH_4_ from aqueous samples in the STRIPS and the release of CH_4_ from a U-trap were thus demonstrated to be effective.

### Method Tests of the CHIPS

The CHIPS had a low and stable blank (±1σ) of 0.67 ± 0.12 μg C and was effective at removing contaminant CO, CO_2_, and C_2_H_6_. Furthermore, the conversion yield (±1σ) of CH_4_ in the CHIPS was high (98.0 ± 1.1%). Combined with effective CH_4_-derived CO_2_ trapping in the final stages of the CHIPS, the combined STRIPS and CHIPS had a high total yield (>90%), as elaborated on in the following sections.

**Figure 3:**
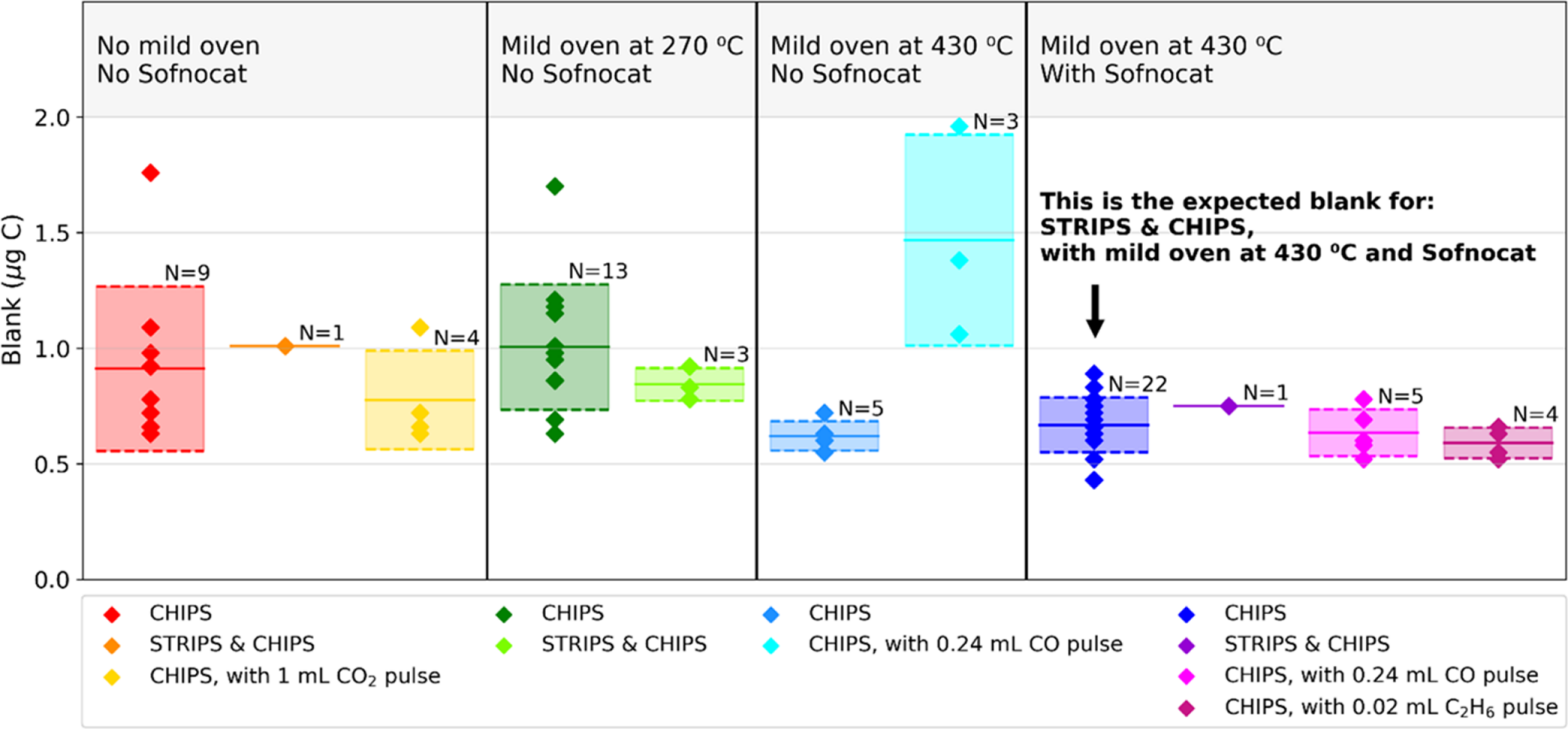
Measured STRIPS and CHIPS blanks for various experimental setups. Horizontal lines show the mean ± 1σ. The dark blue data set (*n* = 22) is the representative blank for the STRIPS and CHIPS combination for the operational protocol designed in this study.

The carbon blank for the CHIPS was found to be small relative to sample signals (Figure 3 and SI Table S10). Specific CHIPS components were addressed separately by including or excluding these in the blank runs (SI Table S8). Including the mild oven (No. 14) at 270 °C did not significantly increase the blank (Figure 3 and SI Table S10). Heating the mild oven to 430 °C reduced the blank to a stable blank of 0.62 ± 0.06 μg C (1σ, *n* = 5). The inclusion of Sofnocat did not increase the carbon blank, and the blank for the CHIPS as described in the operational protocol was thus 0.67 ± 0.12 μg C (1σ, *n* = 22, Figure 3 and SI Table S10). This is similar^14^ or significantly smaller^13,17^ than other vacuum systems aimed at isolating and oxidizing CH_4_ gas from various intermediate sample containers.

Several aspects of the CHIPS operational protocol contributed to the small blank size. Initial pilot tests showed that the blank became ∼3–4 times smaller by adding the CH_4_-scrubber (No. 4 in Figure 2), and the CH_4_-scrubber was therefore incorporated in the CHIPS design. Initial pilot tests with varying gas flows showed that either removing O_2_ or bypassing the main oven substantially decreased the blank (∼9–36 times). Therefore, the CHIPS was designed to minimize both the gas flow through the main oven and the use of O_2_. Furthermore, as initial pilot tests showed that the total flow (rate and time) of both He and O_2_ through the CHIPS influenced the blank size, the described operational steps and their exact timings must be closely followed to guarantee the observed stable and low blank (Figure 3).

The catalysts of the CHIPS were each separately tested and found effective. The conversion yield of CH_4_ in the main oven (No. 16 in Figure 2) of the CHIPS was 98.0 ± 1.1% (1σ, *n* = 4, SI Table S8). A high yield was expected as former methods used platinized quartz wool at ∼700^20^ and 850–900^14^ °C, and we used this catalyst at an even higher temperature (975 °C). Yet, initial pilot tests showed that the quartz tube volume and the amount of catalyst were critical for the combustion yield.

The CO was effectively removed by the combination of the mild oven (No. 14) and Sofnocat (No. 13) while preserving CH_4_. The CRDS-based CO conversion yield in the mild oven of the CHIPS was 101.4 ± 1.3% (1σ, *n* = 3, SI Table S8). Initial pilot tests addressing CO conversion at a lower temperature (∼270 °C) in the mild oven yielded incomplete oxidation of CO (∼84–49% for ∼0.1–0.4 mL CO injections). It is thus important to operate the CHIPS according to the operational protocol with a mild oven heated to ∼430 °C when CO is present in the sample. However, blank experiments with CO pulses, in size double that of the maximum CO amount encountered in our samples, showed that small traces of CO remained after the mild oven (Figure 3 and SI Tables S8 and S10). This showed that only CRDS monitoring, used formerly to assess the conversion and removal of certain C-contaminant gases like CO and NMHCs,^14^ is not an adequate method to determine complete removal down to the smallest remaining carbon traces. The CO pulses were quantitatively removed when also Sofnocat was included (Figure 3 and SI Tables S8 and S10).

The reduction of CH_4_ by the mild oven of the CHIPS was 2.1 ± 1.6% (1σ, *n* = 3, SI Table S8). The combination of mild oven and Sofnocat reduced CH_4_ by 0.1 ± 1.0% (1σ, *n* = 3, SI Table S8). It is therefore recommended to include Sofnocat when large amounts of CO are observed in samples, as it effectively removes CO while not significantly affecting the CH_4_ or increasing the blank. Taken together, any trace CO was quantitatively removed by the mild oven and Sofnocat while, importantly, CH_4_ was not significantly affected.

Similar to the CO removal in the CHIPS, the ability to remove CO_2_ and C_2_H_6_ was tested. Test pulses of CO_2_ were effectively removed in the CHIPS (Figure 3 and SI Tables S8 and S10). The amount of CO_2_ in these pulses was double the amount of CO_2_ that could enter the CHIPS, based on GC-FID analyses of headspace gas in the STRIPS. Initial pilot experiments with CRDS monitoring showed that the VC-6 trap (No. 15 in Figure 2) was critical for effective (∼100%) CO_2_ removal in the CHIPS at the intended total flow rate of ∼2 LPM. A ∼1.6 times smaller VC-4 trap trapped CO_2_ insufficiently (∼87%). Test pulses of C_2_H_6_ were also effectively removed in the full CHIPS setup (Figure 3 and SI Tables S8 and S10). The amount of C_2_H_6_ in these pulses was set to double the maximum amount of C_2_H_6_ encountered in East Siberian Arctic Shelf samples. As CRDS monitoring cannot prove the total removal of NMCs down to the smallest traces, we suggest that it is important to test the system by injecting pulses of CO_2_, CO, and C_2_H_6_ into the vacuum line when constraining the carbon blank. The CHIPS has thus both a very stable and low blank and is more thoroughly tested for removing NMCs than any earlier published method for isolating CH_4_ from aqueous samples for ^14^C diagnostics.

The CHIPS system was also constrained for the natural abundance radiocarbon (Δ^14^C) signal of the blank (SI Table S8) as this facilitates any putative Δ^14^C–CH_4_ blank correction. The measured Δ^14^C (±1σ) for the set of pooled blanks (*n* = 11) for the CHIPS operated without Sofnocat and varying mild oven temperatures was −231 ± 141‰ (SI Table S8). The measured Δ^14^C (±1σ) for the set of pooled blanks (*n* = 17) produced when operating the CHIPS exactly according to the operational protocol was −355 ± 13‰ (SI Table S8). As the next section will show that the STRIPS blank is negligible, the blank Δ^14^C (±1σ) value of −355 ± 13‰ is considered the total blank Δ^14^C value for our described STRIPS and CHIPS operational protocols.

### Full-System Blank of the STRIPS and the CHIPS

The full-system blank was small (0.67 ± 0.12 μg C) in context of the objective of establishing a method suitable for small CH_4_ amounts (down to 10 μg C). The full-system blank is also much lower and more stable than earlier reported methods; this is one of the main overall improvements with the developed method. A low and stable blank with respect to sample size is next to a low uncertainty in the blank Δ^14^C value, the most important factor for achieving a low uncertainty in the blank-corrected sample Δ^14^C–CH_4_ value, which in turn improves the CSRA-based source apportionment of CH_4_.

The carbon background was constrained for the combination of the STRIPS and CHIPS (Figure 3 and SI Table S10), and it was observed that the STRIPS does not add any significant carbon (<0.1 μg C) above that of the CHIPS blank for any of the combined STRIPS and CHIPS setups tested. This means that the cap change, headspace creation, and operation of the STRIPS involved less than ∼100 mL ambient air leakage (representing ∼0.1 μg ambient C–CH_4_) into the sample. Our method of headspace creation differs from former methods where the headspace was achieved by filling underpressurized He-containing kegs^13^ or evacuated carboys^17^ partly with seawater. Storing sample containers without a gas phase and using CH_4_-scrubbed He for the headspace creation are improvements as this limits contamination by nonsample CH_4_, likely contributing to the observed low carbon blank. Furthermore, the effect of the storage time of a U-trap on the carbon blank was assessed (up to 218 days, as shown in SI Table S10), and no measurable effect was found. The U-traps used to evaluate the full-system blank were run as second, third, or fourth in a daily CHIPS sequence as (up to three) actual samples are run after one initial blank run. The full-system blanks did not increase with the position in the CHIPS sample sequence (SI Table S10). Taking it all together, the carbon background for the CHIPS can thus be used as the total blank (±1σ) for the STRIPS and the CHIPS, i.e., 0.67 ± 0.12 μg C.

A full-system blank (±1σ) of 0.67 ± 0.12 μg C is 7–9 times lower than reported total blanks of earlier methods.^14,17^ Hence, the current method is an important improvement relative to existing methods as it facilitates CSRA of CH_4_ in ambient water samples that are much smaller. It is unclear in which hardware and/or operational parts the largest advantages lie relative to earlier systems with higher carbon blanks, as many aspects are different. Nevertheless, it is deduced that one important part of the overall methodological advance likely comes from the extraction phase with the STRIPS compared to other stripping methods.^13,14,17^ Two main improvements might originate from operating the STRIPS at overpressure, limiting ambient air intrusion, and the headspace-sample equilibration phase to maximize CO_2_ removal before CH_4_ trapping in the U-trap. We observed a significant pressure drop in our system when cooling the U-trap. This might similarly have lowered initial (sub-) ambient pressures in similar systems^13,17^ even further. Our method avoids sample compression into a gas cylinder, which would cause a high blank.^14^ Considering the much lower full-system blank for our combined STRIPS and CHIPS compared to former methods, the current system is a major improvement for CSRA of CH_4_ in ambient water.

### Methane Recovery in the STRIPS and the CHIPS

**Figure 4:**
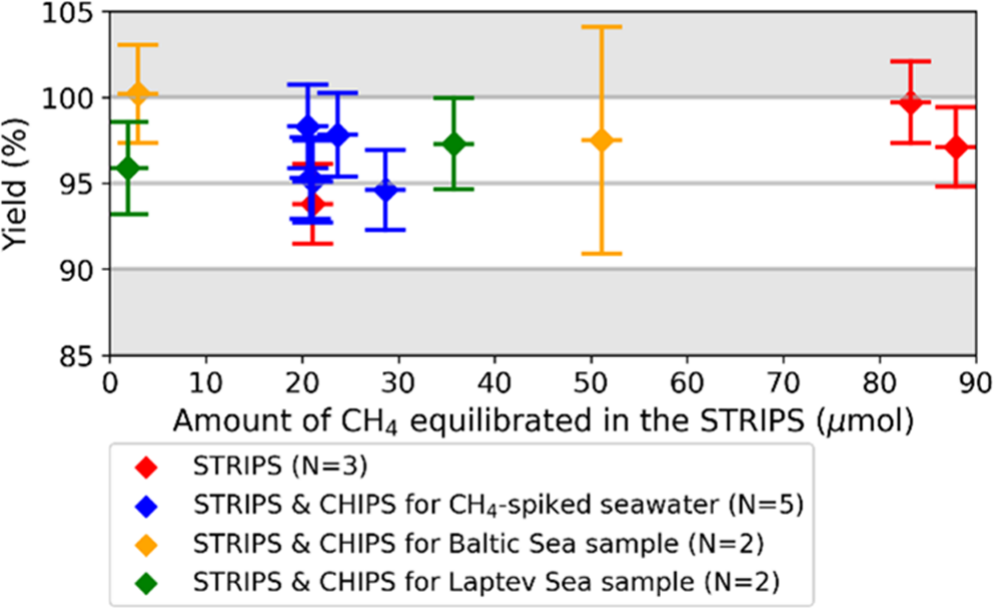
Yield (±1σ) of the STRIPS and the combination of the STRIPS and the CHIPS for both CH_4_-spiked and ambient water samples.

High CH_4_ recoveries were found for the combined STRIPS and CHIPS. Both CH_4_-spiked seawater and samples with ambient CH_4_ always showed a >90% yield, on average 96.9 ± 1.8% (1σ, *n* = 9) (Figure 4 and SI Tables S9 and S11). Storage times of the U-trap between the operation of the STRIPS and the CHIPS over 13–323 days did not affect the measured yields (SI Table S11). This demonstrates the feasibility of executing the STRIPS part in the field to reduce the water samples to the much smaller U-traps, greatly facilitating the transport of samples to laboratory-based CHIPS analytical facilities. The applied temperature of the mild oven (No. 14 in Figure 2) did not affect the measured yield (SI Table S11), which is consistent with the insignificant reduction of CH_4_ by the mild oven and Sofnocat (SI Table S8). Furthermore, the combined STRIPS and CHIPS have, after 67 samples over 19 months, not shown any degradation in the performance of adsorbents or catalysts.

The combined yield of the STRIPS and the CHIPS was similar in size to the yield of the STRIPS only (Figure 4), which thus implies that the CH_4_ conversion in the CHIPS is very effective, consistent with the CRDS-based CH_4_ conversion yield (SI Table S8). The high yield also indicates effective CO_2_ trapping in the LN_2_-cooled VC-6 trap (No. 18 in Figure 2). As no CO was observed for the CH_4_-spiked seawater or Baltic Sea samples, the mild oven was kept at 270 °C for some samples (SI Table S11) while considering the relevant blank value (Figure 3 and SI Table S10). The Laptev Sea samples had a non-negligible CO amount, yet much lower than the tested 0.24 mL (SI Table S8). Therefore, analyzing these samples without Sofnocat (SI Table S11) could have increased the blank for these runs by maximally 0.85 ± 0.46 μg (±1σ, SI Table S10), reducing the yield by maximally 0.2 and 3.9%, for the large and small Laptev Sea samples, respectively. In addition to high yields of the combined STRIPS and CHIPS for spiked and natural samples, the observed yields were also constant for a wide range of sample amounts of 2–51 μmol CH_4_ (Figure 4). These correspond to seawater CH_4_ concentrations of 90–2400 nM, which are representative for CH_4_ hotspot areas. Conclusively, a high total recovery was constrained for different samples and the here developed STRIPS and CHIPS.

### Demonstration of the Entire System to Constrain the Radiocarbon Signal of Aqueous Methane

The Δ^14^C value of sample CH_4_ was observed to be stable throughout the consecutive STRIPS and CHIPS operations and was also determined for ambient field samples. The CH_4_ isolated from the spiked seawater samples showed after the STRIPS and the CHIPS a Δ^14^C–CH_4_ value equally depleted as the spiking material (Δ^14^C–CH_4_ <−997‰) for a variety of U-trap storage times and inclusion of a 50 mL ambient air spike (Table 1). This means that the operation of the combined STRIPS and CHIPS and U-trap storage did not add any significant ambient air carbon contamination, as such contamination would have a close to modern Δ^14^C–CH_4_. This natural abundance radiocarbon result was consistent with our very low carbon background (Figure 3).

The observed natural abundance Δ^14^C–CH_4_ values for ambient water samples varied from ^14^C-depleted CH_4_ for the Laptev Sea to more modern CH_4_ for the Baltic Sea (Table 1). This suggests microbially produced CH_4_ from recently accumulated organic matter in the anoxic Landsort Deep basin sediments of the Baltic Sea. In contrast, the Outer Laptev Sea in the Arctic Ocean showed a fully ^14^C-depleted signal, suggesting either CH_4_ originating from very old subsea permafrost/CH_4_ hydrates or an old natural gas reservoir, as was earlier suggested by a limited-scale triple-isotope study.^13^ In contrast, the Inner Laptev Sea sample tested here for method applicability showed a Δ^14^C–CH_4_ (±1σ) value of −828.1 ± 11.4‰ (Table 1). For this documented CH_4_ hotspot region,^18^ this is the first seawater Δ^14^C–CH_4_ measurement. The sample Δ^14^C–CH_4_ value corresponds to a radiocarbon age of ∼14.1 ky BP, slightly older than the time of inundation at this location (∼9 ky BP).^8,22^ The Δ^14^C–CH_4_ measurement could, therefore, point toward microbial CH_4_ from thawing subsea permafrost. It may also represent an average radiocarbon signal of CH_4_ being a mixture of older and younger CH_4_ sources. The developed method enables measuring the natural abundance radiocarbon fingerprint of CH_4_ in natural waters and may, combined with stable isotope analysis of hydrogen and carbon in CH_4_, provide insights into which sources emit CH_4_ to the Arctic Ocean and the overlying atmosphere and similarly be applied to any aqueous CH_4_ hotspot elsewhere in the world.

**Table 1:** Sample Types, Experimental Setups, and Radiocarbon Analysis Results for the Demonstration of the Intended Application of the STRIPS and the CHIPS to Constrain the Source-Diagnostic Radiocarbon Signal of Elevated CH_4_ in Natural Waters

sample type	sample location and depth	50 mL ambient air injected	U-trap storage time (days)	sealed ampule	blank-corrected Δ^14^C ± 1σ (‰)
CH_4_-spiked seawater		no	97	not produced	
CH_4_-spiked seawater		yes	19	yes	technical malfunction at AMS facility
CH_4_-spiked seawater		yes	20	yes	<−999.6
CH_4_-spiked seawater		no	323	yes	<−999.3
CH_4_-spiked seawater		yes	218	yes	<−998.8
Baltic Sea sample	58.6°N, 18.2°E, 150 m		13	yes	15.4 ± 8.8
Baltic Sea sample	58.6°N, 18.2°E, 430 m		14	broken ampule	
Inner Laptev Sea sample	73.1°N, 130.5°E, 21 m		176	yes	–828.1 ± 11.4
Outer Laptev Sea sample	76.8°N, 125.9°E, 66 m		174	yes	<−998.5

## Conclusions

An effective two-stage preparation system (STRIPS and CHIPS) for radiocarbon analysis of CH_4_ dissolved in seawater and other aqueous media like freshwater has been developed. This new system allows powerful source-diagnostic Δ^14^C–CH_4_ determination of quickly sampled 30 L aqueous samples with CH_4_ concentrations down to 40 nM. Analyzing aqueous samples with even lower CH_4_ concentrations may be possible by processing multiple sample containers in series with the STRIPS and collecting the extracted CH_4_ in a single U-trap. With slight modifications of our subsystem CHIPS, this system may also be used for Δ^14^C–CH_4_ determination of atmospheric samples being prepared to have the O_2_ partial pressure in the CHIPS lower than the vapor pressure of O_2_ at LN_2_ temperature. This system can possibly also be further developed to prepare aqueous and atmospheric CH_4_ for analysis of doubly substituted isotopologues of CH_4_ (^13^CH_3_^2^H and CH_2_^2^H_2_) after constraining any fractionation of ^13^C and ^2^H. Modifications for this application would include bypassing the main oven and adding a method for trapping purified CH_4_.

The combination of the STRIPS and the CHIPS is demonstrated to have a robustly high yield, a consistently low and well-defined blank, allowing analysis of much smaller sample sizes (down to 10 μg C–CH_4_) than earlier approaches. In combination with the demonstrated stability of Δ^14^C–CH_4_ in the STRIPS and the CHIPS and its successful application to real samples from the Baltic and Arctic Seas, our established CSRA method can be used to study sources of released CH_4_ from the subsea permafrost system on the shallow and extensive Arctic shelf seas. The developed method for source-diagnostic CSRA of aqueous CH_4_ can be further applied to (SI Table S14): other seas, wetlands and lakes across the Arctic and in the tropics, and other natural and artificial aquatic systems elsewhere, especially where uncertainties in natural and/or anthropogenic emissions are high.

## Supporting Information Available

Pictures of the STRIPS (Figures S1.1–S1.3); pictures of the CHIPS (Figures S2.1–S2.5); construction parts of the STRIPS and the CHIPS (Tables S1–S2); detailed preparation and operational procedures of the STRIPS and the CHIPS (Tables S3–S6); details regarding the STRIPS and CHIPS method tests (Tables S7–S9); experimental setups and results of individual tests: blanks of the CHIPS, blanks and yields of the STRIPS and the CHIPS (Tables S10–S11); in-field sampling procedures (Table S12); isolated headspace creation system (Figure S3); comparison of methods (Table S13); global range to apply the STRIPS and the CHIPS (Table S14) (PDF)
