# Supplementary material for: Isolation of Methane from Ambient Water and Preparation for Source-Diagnostic Natural Abundance Radiocarbon Analysis
Source: Anal Chem. 2024 Oct 24;96(44):17631–9. doi: 10.1021/acs.analchem.4c03525 (PMC11541896; doi:10.1021/acs.analchem.4c03525)
Supplement: Supplementary file 1 — ac4c03525_si_001.pdf [file ac4c03525_si_001.pdf]

Isolation of Methane from Ambient Water and Preparation for Source-Diagnostic Natural Abundance Radiocarbon Analysis

Marenka Brussee[ab*], Henry Holmstrand[ab], Michael Süß[ab], Amelia Davies[ab†], and Örjan Gustafsson[ab*]

[a]*Department of Environmental Science, Stockholm University, Stockholm 10691, Sweden*

[b]*Bolin Centre for Climate Research, Stockholm University, Stockholm 10691, Sweden*

Corresponding Authors

[*]M.B.: Email: Marenka.Brussee@aces.su.se
[*]Ö.G.: Email: Orjan.Gustafsson@aces.su.se

Present Address

[†]A.D.: Institute for Geology and Mineralogy, University of Cologne, Cologne 50674, Germany

SUPPORTING INFORMATION

Contents

1. Pictures of the STRIPS. S4

Figure S1.1: The STRIPS: Stripping board connected to keg. S4

Figure S1.2: Customized headpiece. S5

Figure S1.3: Customized U-trap. S5

2. Pictures of the CHIPS. S6

Figure S2.1: The CHIPS, including the sample U-trap (left arrow) and final glass ampule (right arrow). S6

Figure S2.2: Manometric assembly for manometric quantification (middle) and glass ampule (right). S6

Figure S2.3: The VC-6 trap (No. 15 and 18 in Figure 2). S7

Figure S2.4: The design of the VC-6 trap (No. 15 and 18 in Figure 2). S7

Figure S2.5: The LN_2-elevator system used during the sub-transfer of CO_2 from the VC-6 trap (No. 18 in Figure 2) to the VC-2 trap (No. 19 in Figure 2). S8

3. Construction parts of the STRIPS and the CHIPS. S9

Table S1. Construction parts of the STRIPS. S9

Table S2: Construction parts of the CHIPS. S12

4. Detailed preparation and operational procedures of the STRIPS and the CHIPS. S16

Table S3: Details of preparation steps of the STRIPS. S16

Table S4: Operational procedure of the STRIPS for blank and sample runs. S18

Table S5: Details of preparation steps of the CHIPS. S19

Table S6: Operational procedure of the CHIPS for blank and sample runs. S21

5. Details regarding the STRIPS and CHIPS method tests. S27

Table S7: Details regarding specific method tests of the STRIPS. S27

Table S8: Details regarding specific method tests of the CHIPS. S29

Table S9: Details regarding specific methods for testing the STRIPS and CHIPS combination. S32

6. Experimental setups and results of individual tests: blanks of the CHIPS, blanks and yields of the STRIPS and the CHIPS. S33

Table S10: Specific experimental details and individual blanks of the CHIPS and the combination of the STRIPS and the CHIPS. S33

Table S11: Specific experimental details and yields for the combination of the STRIPS and the CHIPS. S36

7. In-field sampling procedures. S37

Table S12: Procedures of filling sampling kegs. S37

8. Isolated headspace creation system. S39

Figure S3: Flowchart of an isolated headspace creation system in the field. S39

9. Comparison of existing methods and the STRIPS and the CHIPS. S40

Table S13: Comparison of existing methods for CH_4 extraction from ambient water and subsequent quantitative conversion to CO_2 for compound-specific radiocarbon analysis of CH_4 versus the STRIPS and the CHIPS. S40

10. Global applicability of the STRIPS and the CHIPS for radiocarbon-based source apportionment of CH_4 in ambient water. S43

Table S14: Examples of global systems (ocean regimes and inland systems) where a CH_4 range has been reported to make it feasible to apply the STRIPS and the CHIPS for compound-specific radiocarbon analysis of CH_4. .... S43

11. References S46

1. Pictures of the STRIPS.

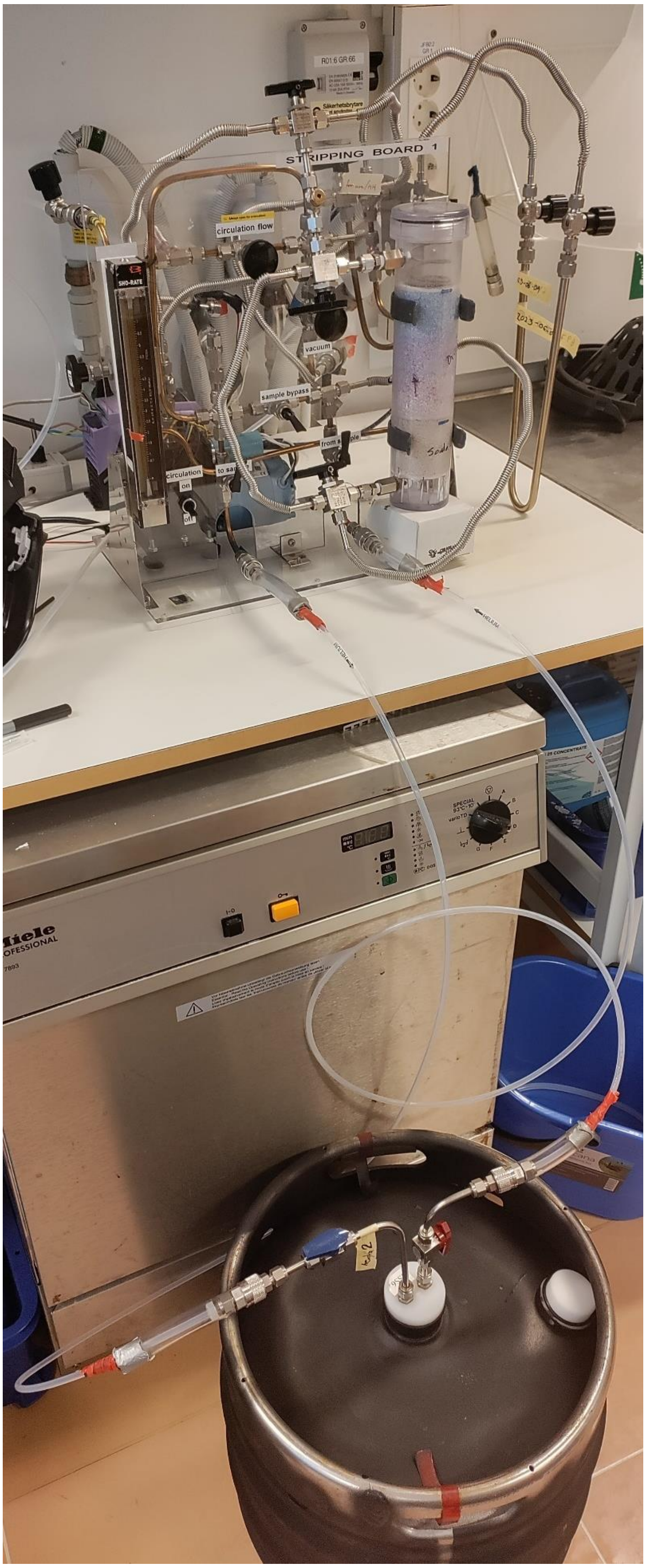

Figure S1.1: The STRIPS: Stripping board connected to keg.

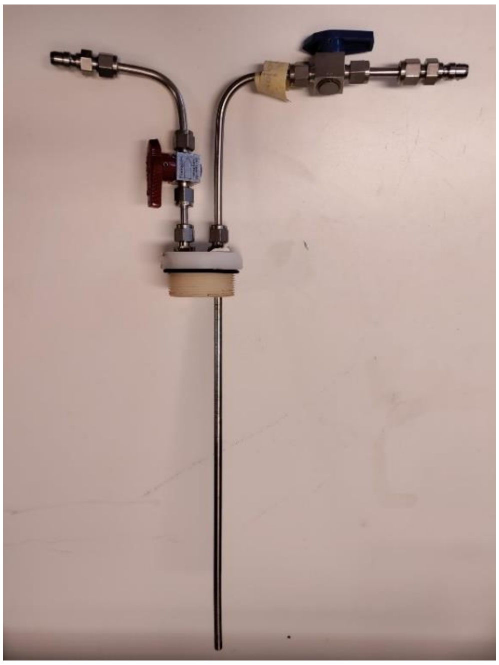

Figure S1.2: Customized headpiece.

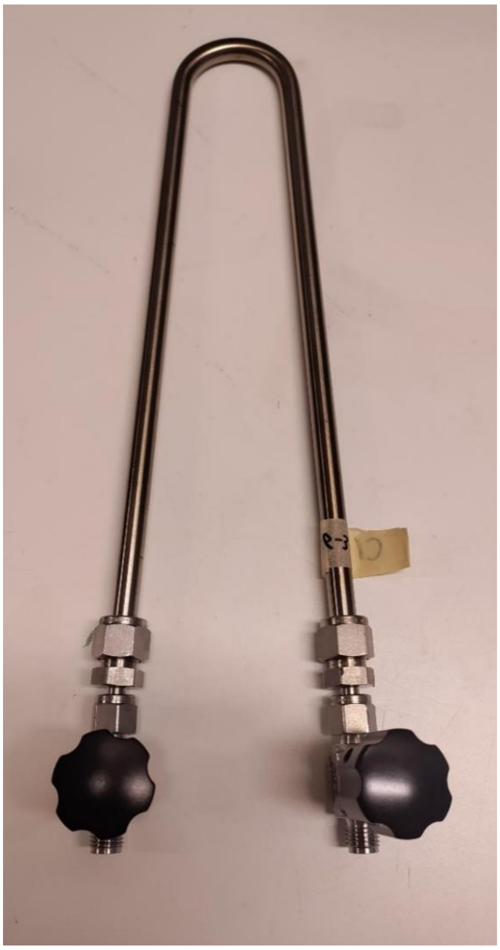

Figure S1.3: Customized U-trap.

2. Pictures of the CHIPS.

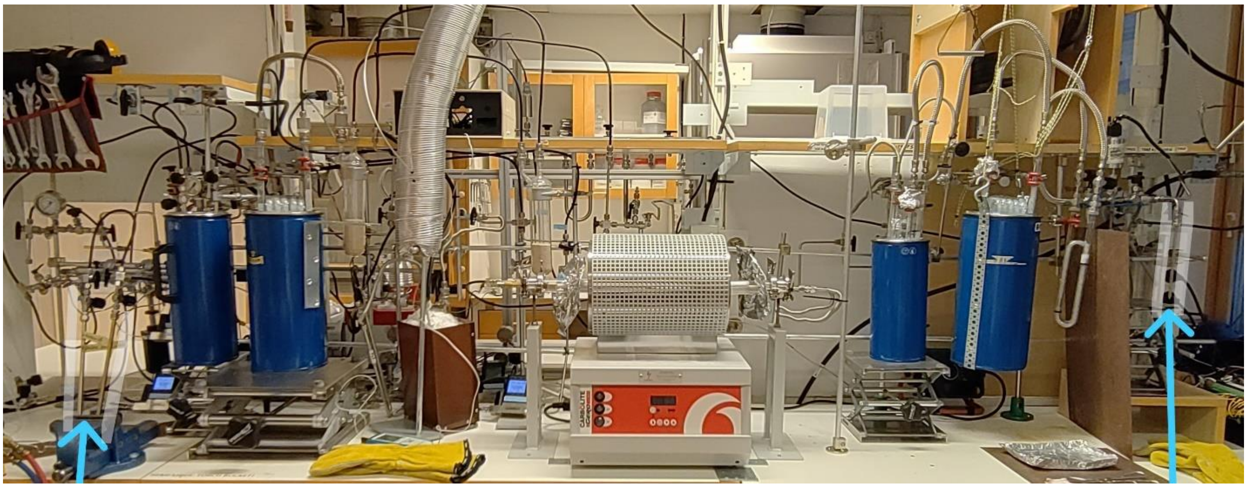

Figure S2.1: The CHIPS, including the sample U-trap (left arrow) and final glass ampule (right arrow).

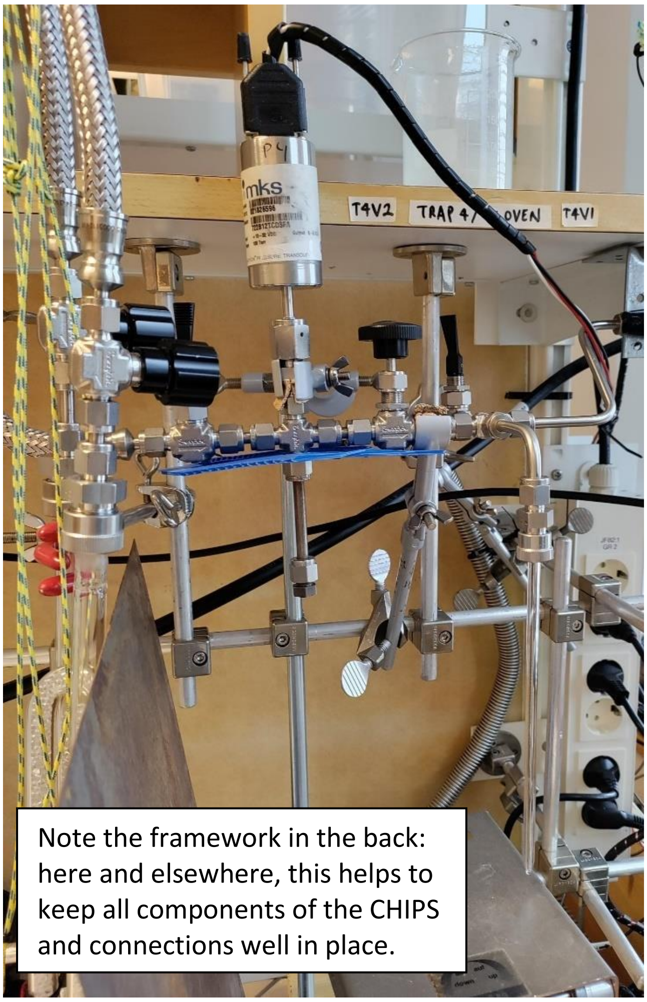

Figure S2.2: Manometric assembly for manometric quantification (middle) and glass ampule (right).

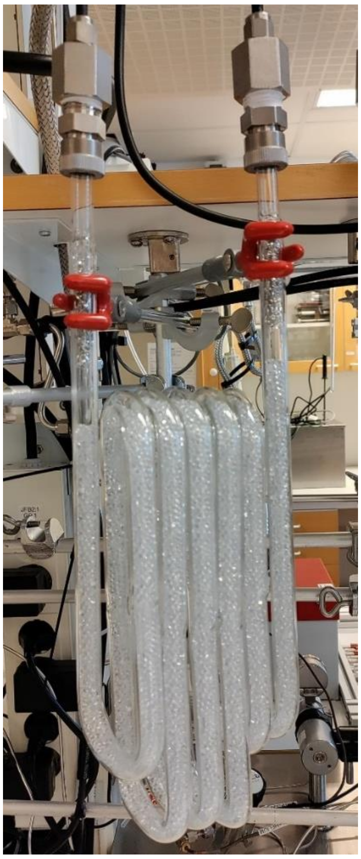

Figure S2.3: The VC-6 trap (No. 15 and 18 in Figure 2).

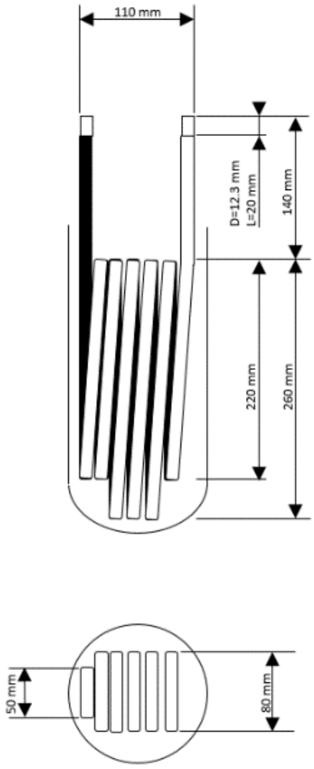

Figure S2.4: The design of the VC-6 trap (No. 15 and 18 in Figure 2).

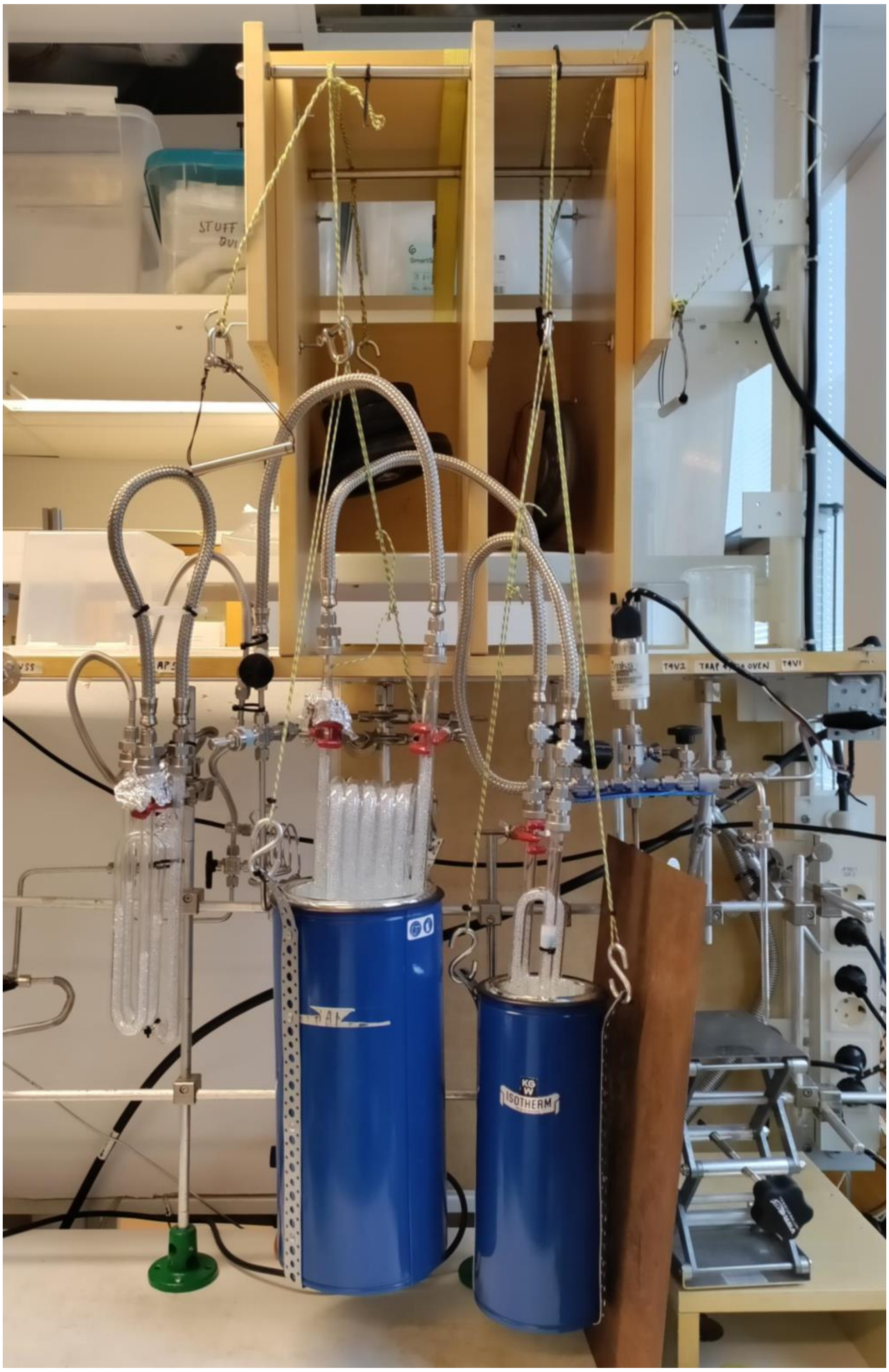

Figure S2.5: The LN_2-elevator system used during the sub-transfer of CO_2 from the VC-6 trap (No. 18 in Figure 2) to the VC-2 trap (No. 19 in Figure 2).

3. Construction parts of the STRIPS and the CHIPS.

Table S1. Construction parts of the STRIPS.

Item	No. in the STRIPS flowchart (Figure 1)	Product number/name	Manufacturer/ supplier	Additional notes
**Stainless steel 1:4 in. tubing for stripping board**		CTX21515	Dalco Metals, Inc. - Walworth, WI, U.S.A.	
**Flexible bellows 1:4 in. tubing for stripping board**			Swagelok Sweden – Sollentuna, Sweden	321 Stainless Steel.
**All nuts and ferrules**			Swagelok Sweden – Sollentuna, Sweden	
**All valves (toggle, needle, and 3-way valves)**	11, 12, 16		Swagelok Sweden – Sollentuna, Sweden	
**30 L stainless-steel keg**	1	EuroKeg	BLEFA GmbH – Kreuztal, Germany	
**Custom-made polyoxymethylene (POM) lid**	2 (part of headpiece as well as storage lid)		PROFILPLAST AB – Mörarp, Sweden	Material: POM natur.
**O-ring for the custom-made lid**	2 (part of headpiece as well as storage lid)		LAMISA Teknik AB – Vällingby, Sweden	Size: 49.5x3.0mm. Material: NBR70.
**Stainless steel 1:4 in. tubing for headpiece**	2 (part of headpiece)	CTX21515	Dalco Metals, Inc. - Walworth, WI, U.S.A.	
**Tube fitting screwed in POM lid (male NPT fitting screwed into lid)**	2 (part of headpiece)	#SS-400-1-4-BT	Swagelok Sweden – Sollentuna, Sweden	Fittings were drilled through with a press drill to allow 1:4 in. tubing through the fitting. Twice used in construction headpiece.
**Red quarter turn plug valve**	2 (part of headpiece)	#SS-4P4T-RD	Swagelok Sweden – Sollentuna, Sweden	Once used in construction headpiece.
**Blue quarter turn plug valve**	2 (part of headpiece)	#SS-4P4T-BL	Swagelok Sweden – Sollentuna, Sweden	Once used in construction headpiece.
**Quick connect stem**	2 (part of headpiece)	#SS-QF4-S-400	Swagelok Sweden – Sollentuna, Sweden	Twice used in construction headpiece.

Item	No. in the STRIPS flowchart (Figure 1)	Product number/name	Manufacturer/ supplier	Additional notes
**180°-bent 61 cm long 3:8 in. tubing**	3 (U-trap component)		Swagelok Sweden – Sollentuna, Sweden	316/316L Stainless Steel.
**HiSiv 3000**	3 (U-trap component)		Kurt Obermeier GmbH - Bad Berleburg, Germany	Particle format: 1:16 in. rods. The HiSiv 3000 is held in the U-trap with stainless steel wool on each side.
**Swagelok tube adapter**	3 (U-trap component)	#SS600R4	Swagelok Sweden – Sollentuna, Sweden	Twice used in construction U-trap.
**Needle valve**	3 (U-trap component)	#SS14DKS4	Swagelok Sweden – Sollentuna, Sweden	Twice used in construction U-trap.<br>It can handle up to 207 bar at 37 ^{o}C.
**Acrylic gas purifying unit**	5	#26848 L68GP	W. A. Hammond DRIERITE Co. LTD – Xenia, OH, U.S.A.	This unit contains 300 g indicating Drierite (10-20 mesh) and 100 g soda lime, separated by felt filters.
**Felt filters**	5 (part of gas purifying unit)	Z248444	Sigma Aldrich – Saint Louis, MO, U.S.A.	
**Circulation pump**	6	MB-21E PUMP/COMPRESSOR W/TEFLON VALVE GASKET P#48725 S#20615	Senior Metal Bellows - Sharon, MA, U.S.A.	
**Vacuum pump**	7	#N145.1.2AT.18	KNF Neuberger GmbH - Freiburg im Breisgau, Germany	
**Pressure transducer**	9	#CTE8002AM7V	Sensortechnics GmbH – Puchheim, Germany	Pressure range: 0-2 bar. Accuracy: ±0.1 %FSO. This pressure transducer is connected to a CR1000 Campbell Scientific datalogger.

Item	No. in the STRIPS flowchart (Figure 1)	Product number/name	Manufacturer/ supplier	Additional notes
**Flowmeter**	10	Sho-Rate GT 355/D-2-R-2-15-C-E3-D-1-A-0-0-0-2	TemFlow Control AB - Stockholm Sweden	
**Quick connect fitting**	13	#SS-QF4-B-400	Swagelok Sweden – Sollentuna, Sweden	
**Flexible 1:4 in. Perfluoroalkoxy (PFA) tubing**	14		Swagelok Sweden – Sollentuna, Sweden	Material: PFA plastic. Wall thickness: 0.062 in. OD: 1:4 in.
**He* grade 5.0**	15		Strandmöllen AB – Ljungby, Sweden	*In case of helium scarcity, it is expected that helium can be replaced with neon.
**Pressure gauge**		#PGI-63B-BG10-LAQX	Swagelok Sweden – Sollentuna, Sweden	Used for U-trap leakage testing.
**Sintered stainless steel filter**		#SS4FK490	Swagelok Sweden – Sollentuna, Sweden	Connected to the longest tube of the custom-made headpiece of the STRIPS (No. 2 in Figure 1) for blank water creation.

Table S2: Construction parts of the CHIPS.

Item	No. in the CHIPS flowchart (Figure 2)	Product number/name	Manufacturer/ supplier	Additional notes
**Stiff 1:4 in. tubing for construction of the CHIPS**			Swagelok Sweden – Sollentuna, Sweden	316/316L Stainless Steel.
**Flexible bellows 1:4 in. tubing for construction of the CHIPS**			Swagelok Sweden – Sollentuna, Sweden	321 Stainless Steel.
**Flexible 1:4 in. Synflex tubing for construction of the CHIPS**		Synflex Type “1300”	Eaton Corporation plc – Beachwood, OH, U.S.A.	
**All nuts and ferrules**			Swagelok Sweden – Sollentuna, Sweden	
**All valves (toggle and 3-way valves)**	3, 5		Swagelok Sweden – Sollentuna, Sweden	
**Ultra-Torr fittings**	Part of 11, 14, 15, 17, 18, 19, 22		Swagelok Sweden – Sollentuna, Sweden	Used to connect 1:4 in. tubing to glass.
**He* grade 5.0**	1		Strandmöllen AB – Ljungby, Sweden	*In case of helium scarcity, it is expected that helium can be replaced with neon.
**Mass flow controller**	2	MFC FMA-2607A	Omega Engineering, Inc. - Norwalk, CT, U.S.A.	
**HiSiv 3000**	4 (part of CH_4-scrubber)		Kurt Obermeier GmbH - Bad Berleburg, Germany	Particle format: 1:16 in. rods.
**180°-bent 61 cm long 3:8 in. tubing**	4 (part of CH_4-scrubber)		Swagelok Sweden – Sollentuna, Sweden	316/316L Stainless Steel.
**180°-bent 61 cm long 3:8 in. tubing**	6 (U-trap component)		Swagelok Sweden – Sollentuna, Sweden	316/316L Stainless Steel.

Item	No. in the CHIPS flowchart (Figure 2)	Product number/name	Manufacturer/ supplier	Additional notes
**HiSiv 3000**	6 (U-trap component)		Kurt Obermeier GmbH - Bad Berleburg, Germany	Particle format: 1:16 in. rods.
**Swagelok tube adapter**	6 (U-trap component)	#SS600R4	Swagelok Sweden – Sollentuna, Sweden	Twice used in construction U-trap.
**Needle valve**	6 (U-trap component)	#SS14DKS4	Swagelok Sweden – Sollentuna, Sweden	Twice used in construction U-trap. It can handle up to 207 bar at 37 ^{o}C.
**Quick connect fitting**	7	#SS-QF4-B-400	Swagelok Sweden – Sollentuna, Sweden	
**Expansion volume**	9	#316L-HDF4-500CC	Swagelok Sweden – Sollentuna, Sweden	Volume: 0.5 L.<br>Material: 316L Stainless Steel.<br>It can handle up to 124 bar.
**Vacuum pump**	10	MD1	Vacuubrand GmbH - Wertheim, Germany	
**“Russian doll” trap**	11		Labglas AB – Stockholm, Sweden	Material: borosilicate glass.<br>Size: ~0.8 L internal volume.
**Zeolite “13X APG”**	11 (part of CO_2-scrubber)	UOP Molekularsieb 13X APG 8x12	Kurt Obermeier GmbH - Bad Berleburg, Germany	
**Gas mixture: 80% He* grade 6.0 and 20% O_2 grade 5.2**	12		Linde Gas AB – Solna, Sweden	*In case of helium scarcity, it is expected that helium can be replaced with nitrogen.
**Sofnocat**	13 (part of CO removal trap)	Sofnocat 423	Molecular Products Ltd - Harlow, United Kingdom	25 g Sofnocat is loaded in the 3:8 in. stainless steel tube.
**180°-bent 61 cm long 3:8 in. tubing**	13 (part of CO removal trap)		Swagelok Sweden – Sollentuna, Sweden	316/316L Stainless Steel.

Item	No. in the CHIPS flowchart (Figure 2)	Product number/name	Manufacturer/ supplier	Additional notes
**Custom-made mild oven**	14			Construction: Borosilicate glass tube (VC-2 trap) for 4/9th filled with CuO, glass wool insulated and heated with heating tape. The VC-2 trap has 2 coils and a total path length of 93 cm.
**CuO**	14 (catalyst in mild oven)		Merck KGaA - Darmstadt, Germany	Wire fine: ~0.65x3 mm.
**Mild oven PID controller**	14 (temperature controller in mild oven)	CNi3233	Omega Engineering, Inc. - Norwalk, CT, U.S.A.	
**VC-6 trap**	15 and 18		Labglas AB – Stockholm, Sweden	Material: borosilicate glass.<br>Size: 6 coils, total path length 335 cm.<br>Filling: 3 mm glass beads.<br>Further details are shown in Figure S2.4.
**Main oven**	16	MTF 12/38/250 # M1238-250-208SN	Carbolite Gero GmbH & Co. KG – Neuhausen, Germany	
**Quartz tube**	16 (part of CH_4 oxidation oven)	#TU-QTZ-029-0600	Carbolite Gero GmbH & Co. KG – Neuhausen, Germany	ID: 29 mm. OD: 32 mm. L: 600 mm.
**End seal 1**	16 (part of CH_4 oxidation oven)	#TS-032-042-0130	Carbolite Gero GmbH & Co. KG – Neuhausen, Germany	Contains O-ring.
**End seal 2**	16 (part of CH_4 oxidation oven)	#TS-032-042-0020	Carbolite Gero GmbH & Co. KG – Neuhausen, Germany	Contains O-ring.
**Insulation plugs (at both sides of tube)**	16 (part of CH_4 oxidation oven)	#51-50-11	Carbolite Gero GmbH & Co. KG – Neuhausen, Germany	For tube OD: 32 mm. Material: Ceramic, Type D.
**Platinized quartz wool**	16 (part of CH_4 oxidation oven)	#630-00996	Shimadzu Corporation – Kyoto, Japan	The oven contains 8 packages of this high sensitivity TC catalyst.

Item	No. in the CHIPS flowchart (Figure 2)	Product number/name	Manufacturer/ supplier	Additional notes
**VC-2 trap**	17 and 19		Labglas AB – Stockholm, Sweden	Material: borosilicate glass.<br>Size: 2 coils, total path length 93 cm.<br>Filling: 3 mm glass beads.
**Pressure transducer**	20	MKS BARATRON 11XX12 #722B12TCD3FA	MKS Instruments, Inc. – Andover, MA, U.S.A.	Pressure range: 0-100 Torr. Accuracy: 0.5% of Reading. This pressure transducer is connected to a CR1000 Campbell Scientific datalogger.
**Manometric assembly**	21		Swagelok Sweden – Sollentuna, Sweden	Construction: the manometric assembly consists of a Swagelok cross piece and stiff 1:4 in. tubing.<br>Material: 316/316L Stainless Steel.<br>Total volume (± 1σ): 5.84±0.09 mL
**Ampule**	22		Labglas AB – Stockholm, Sweden	Material: Borosilicate.<br>Size: O.D.: 6±0.15 mm.<br>Thickness: 1±0.04 mm.
**High-vacuum pump system**	23	HiCube 80 Eco #PM S03 557 Z	Pfeiffer Vacuum GmbH - Aßlar, Germany	Contains both a turbomolecular pump and a backing (diaphragm) pump.
**Pressure transducer**	24	#CTE8002AM7V	Sensortechnics GmbH – Puchheim, Germany	Pressure range: 0-2 bar. Accuracy: ±0.1 %FSO. This pressure transducer is connected to a CR1000 Campbell Scientific datalogger.

4. Detailed preparation and operational procedures of the STRIPS and the CHIPS.

Table S3: Details of preparation steps of the STRIPS.

Preparation component	Action	Procedure
**U-trap***	Leak testing	A U-trap is tested for leakage by filling the U-trap with N_2 gas to 5.5 bar and monitoring the pressure change over time for each side individually. The pressure is monitored with a pressure gauge alternately attached to each side of the U-trap, and a pressure drop of ≤0.1 bar in 7 days is accepted.
**U-trap***	Cleaning	A U-trap is cleaned with a gentle flow of He (grade 5.0) while being heated to 280 ^{o}C in a custom-built oven for 40 minutes.
**Keg**	Headspace creation	To create the headspace, first, a 1 LPM CH_4-scrubbed He (grade 5.0) flow is created by using the front part of the CHIPS (No. 1-7 in Figure 2) while bypassing No. 6 (Figure 2) or by using an isolated headspace creation system (Figure S3). After swiftly replacing the cap of the keg with the customized headpiece (No. 2 in Figure 1), attaching silicon tubing to the long tube of the headpiece, and after ≥ 5 min of 1 LPM He flow through the LN_2-cooled U-trap (No. 4 in Figure 2 / Figure S3), the He flow is increased to 2 LPM for 1 min. The quick-connect fitting (No. 7 in Figure 2 / Figure S3) is then attached to the short tube of the headpiece (No. 2 in Figure 1), and after opening the two plug valves of the headpiece, water is pushed out through the attached silicon tubing at the long end of the headpiece. After ~5.5 L of sample water is pushed out of the keg, the CH_4-scrubbed He flow is reduced to 1 LPM to remove the final ~4 L of sample water from the keg. To stop the outflow of water (when, in total, 9.5 L of water has been displaced), the two plug valves of the headpiece are closed simultaneously. This results in an absolute pressure of ~1.1 bar in the keg.
**STRIPS**	1st leak testing	The STRIPS, including the gas purifying canister and tubing towards a clean U-trap but excluding the tubing towards the keg, is leak checked at 1.5 bar for 10 min where a <1 mbar/min pressure drop is accepted.
**STRIPS**	2nd leak testing	The STRIPS, including the gas purifying canister, tubing towards a clean U-trap and tubing towards the keg, is leak checked at 1.5 bar for 5 min where a <1 mbar/min pressure drop is accepted.

Preparation component	Action	Procedure
**STRIPS**	Cleaning	The main part of the STRIPS, including the gas purifying unit but without the tubing towards U-trap and keg, is cleaned with He (grade 5.0) by three times repeating consecutive steps of evacuation to ~130 mbar (by using the vacuum pump at No. 7 in Figure 1), filling to ~1900 mbar (from No. 15), and circulation (with No. 6) at ~1100 mbar and 2 LPM for 2 minutes. Thereafter, the STRIPS, including the tubing towards U-trap and keg, is thrice evacuated to ~130 mbar and filled to ~1900 mbar followed by evacuation to a final pressure in the system of ~1030 mbar.
**STRIPS**	3rd leak testing	As a preparation for the next STRIPS runs, this 3rd leak test is executed after the last STRIPS run of the day. The STRIPS, including the gas purifying canister, tubing towards the still connected U-trap, tubing towards the keg, headpiece, and keg, is leak checked at 1.5 bar for 14h where a <40 mbar/14 h pressure drop is accepted. This test ensures the leak-tightness of the headpiece and headpiece-keg connection.

When the STRIPS is operated in the field, it is recommended to prepare the U-traps in a laboratory in advance. [*]

Table S4: Operational procedure of the STRIPS for blank and sample runs.

**Time*** **(min)**	**STRIPS part in operation**	**Flow rate (LPM)**	**Additional action**	**Purpose of phase**
**60**	Full system, excluding U-trap	2		Equilibration of CH_4 between gas and liquid phase.
**~15**	Full system, excluding U-trap	2	Sampling of 10 mL headspace gas and analysis with GC-FID.	Determination of CH_4, CO_2, C_2H_6, and CO concentration in the gas phase. The used GC-FID is the Agilent GC System 7890A (G3440A) with an Agilent CP-PoraBOND Q (P/N: CP7355) column and He as carrier gas.
**2**	Full system, excluding U-trap	0	Pressure reading.	Determination of the pressure in the gas phase when the STRIPS is at rest.
**≥60**	Full system, including U-trap (cooled with LN_2)	2		Trapping CH_4 in the cooled U-trap.
**~8**	Full system, including U-trap (cooled with LN_2)	2	Sampling of 10 mL headspace gas and analysis with GC-FID.	Determination of CH_4, CO_2, C_2H_6, and CO concentration in the gas phase. Confirmation whether all CH_4 is trapped.
**2**	Full system, including U-trap (cooled with LN_2)	0	Pressure reading.	Determination of the pressure in the gas phase when the STRIPS is at rest.
**5**	Full system, including U-trap (cooled with LN_2)	0	Closing the U-trap with adjustable pliers.	Ensuring sample collection in the U-trap. The valves are closed sufficiently at the low operational temperature by using adjustable pliers.

**By a combination of continuous Cavity Ring-Down Spectrometer (CRDS)-monitoring (G2201-i Isotopic Analyzer, supplier: Picarro, Inc.) and off-line GC-FID injections, these times are found sufficient for CH_4 equilibration and CH_4 trapping.*

Table S5: Details of preparation steps of the CHIPS.

Preparation component	Action	Procedure
**Ampule**	Cleaning	The sample ampule is prepared by baking the ampule in an oven at 450 ^{o}C for 6 h. Thereafter, the cleaned ampule is stored in aluminum foil before use.
**Ampule**	Leak testing	After a new ampule is attached to the CHIPS, the tail part (No. 21-22) is leak tested for ≥5 min at ~0.02 mbar. The maximum allowed leak rate is 0.04 mbar/5 min.
**CHIPS**	Cleaning	The CHIPS is evacuated at two locations with the diaphragm vacuum pump (No. 10 in Figure 2) overnight and during periods of non-operation. All three-way valves are turned such that all adsorbents (No. 4 and 11), catalysts (No. 13, 14, and 16), and glass-beads-filled traps (No. 15, 17, 18, and 19) are evacuated. During these periods, the ovens (No. 14 and No. 16) are off (non-heated).
**CHIPS**	Leak testing	A leak check at ~1.5 mbar is executed by using the small-range pressure sensor (No. 20 in Figure 2). The full CHIPS, including components No. 4, 11, 13, 14, 15, 16, 17, 18, 19, 21, and 22, are included in this leak test. The maximum allowed pressure increase during 10 min is 0.11 mbar.
**CHIPS**	Cleaning	This preparation step is executed after the leak test of the CHIPS. During ~0.5 h, a He (grade 5.0) flow of 0.05 LPM is lead through the full line (including components No. 4, 11, 13, 14, 15, 16, 17, 18, 19, 21, and 22 in Figure 2) while having the valve towards the diaphragm pump at the end of the CHIPS open. This allows a small flow rate at low pressure through the system. After starting this ~0.5h preparatory phase of small He flow at low pressure, the ovens are turned on and heated to their final set temperatures.
**Mild oven and main oven**	Conditioning	This preparation step is executed for ovens that have reached their set temperatures (430 ^{o}C for the mild oven (No. 14 in Figure 2) and 975 ^{o}C for the main oven (No. 16)). The system (full line including components No. 4, 11, 13, 14, 15, 16, 17, 18, 19, 21, and 22) is first filled with He. After reaching above ambient pressure, the He-flow is turned so that the flow exits at the tail end of the CHIPS. This is followed by a period of 9 min where 1.65 LPM He is combined with 0.4 LPM He (grade 6.0) and O_2 (grade 5.2) mixture (80:20) to condition and bake out the hot ovens. After turning off the 0.4 LPM He and O_2 flow, O_2 is flushed out of the line by continuous purging with 1.65 LPM He for ≥4 min.
**LN_2-cooled CH_4 and CO_2 scrubbers**	Pre-cooling	The CH_4-scrubber (No. 4 in Figure 2) and CO_2-scrubber (No. 15) are cooled in LN_2 for ≥3 min to reach the starting position for a CHIPS run. The upper 5-8 cm of the VC-6 trap is above the LN_2 level, allowing better CO_2 trapping by the presence of consecutive temperature gradients in the trap.

Preparation component	Action	Procedure
**CHIPS**	Preparation for 2nd, 3rd or 4th run	If multiple samples are processed in series during a day, the following preparation is executed: the tail part (No. 18, 19, 21, and 22 in Figure 2) is filled by a He flow through No. 11, 15, and non-cooled No. 17 (thus bypassing No. 13, 14 and 16). When reaching above ambient pressure, the three-way valve after No. 24 is turned to allow the He flow to exit the system and achieve a stable He flow through the system. When a stable flow of 1.65 LPM He is reached, the flow is consequently guided through also No. 13, 14, and 16. Thereafter, the operational procedure (Table S6) is followed from the action at timing 3 min, i.e., cleaning the full CHIPS, especially No. 13, 14, and 16 with potential CO_2 build-up from the previous run.
**CHIPS**	Preparation for rest position during periods of non-operation	After the last run during a daily CHIPS sequence, the following steps are executed: the tail part (No. 18, 19, 21, and 22 in Figure 2) is filled with a flow through No. 11, 15, and non-cooled No. 17 (thus bypassing No. 13, 14 and 16). When reaching above ambient pressure, the three-way valve after No. 24 is turned to allow the He flow to exit the system and achieve a stable He flow through the system. When a stable flow of 1.65 LPM He is reached, the flow is consequently guided through also No. 13, 14, and 16. For ≥4 min He at 1.65 LPM is purged through the CHIPS to clean the line. Then the flow rate is reduced to 0.05 LPM He. Consequently, the flow is guided towards the diaphragm pump at the tail end of CHIPS, quickly followed by opening the valve towards the diaphragm pump. This is to evacuate the CHIPS and clean the system further. The He flow is thereafter stopped. After reaching base pressure, the valve towards the diaphragm pump (at No. 10) is opened so that CHIPS is evacuated from both ends. When reaching a stable low pressure, after ~5 minutes, the LN_2 cooling baths are removed from the CH_4-scrubber (No. 4) and CO_2-scrubber (No. 15). Thereafter, the ovens are turned off.

Table S6: Operational procedure of the CHIPS for blank and sample runs.

**Time from start* (min)**	**CHIPS part in operation (numbers relate to elements of the CHIPS in Figure 2)**	**Flow in operational part**	**CHIPS part in recovery**	**Flow in recovery part**	**Additional action**	**Purpose of phase**
**0**	Line through No. 11, 13, 14, 15, 16, 17, 18, 19, 21, 22	1.65 LPM He (grade 5.0)	-	-	-	Cooling of CH_4-scrubber (No. 4) and CO_2-scrubber (No. 15). Note: this is the last step of the preparation steps.
**3**	Line through No. 11, 13, 14, 15, 16, 17, 18, 19, 21, 22	1.65 LPM He	-	-	-	Cleaning the full CHIPS, especially No. 13, 14, and 16 with potential CO_2 build-up, potentially from a former run.
**7**	Line through No. 11, 15, 17, 18, 19, 21, 22	1.65 LPM He	-	-	Cool H_2O-scrubbers (No. 17) with an ethanol and dry ice slurry.	H_2O removal from the flow before cooling the CH_4-derived CO_2 collector (No. 18).
**11**	Line through No. 11, 15, 17, 18, 19, 21, 22	1.65 LPM He	-	-	Cool large CH_4-derived CO_2 collector (No. 18).	Preparing large CH_4-derived CO_2 collector (No. 18) for CH_4-derived CO_2 collection.
**15**	Line through No. 11, 13, 14, 15, 16, 17, 18, 19, 21, 22	1.65 LPM He + 0.4 LPM He(80%, grade 6.0)+O_2(20%, grade 5.2)	-	-	-	Supplying No. 13, 14, and 16 with O_2 before sample arrival.

**Time from start[*] (min)**	**CHIPS part in operation (numbers relate to elements of the CHIPS in Figure 2)**	**Flow in operational part**	**CHIPS part in recovery**	**Flow in recovery part**	**Additional action**	**Purpose of phase**
**16**	Line through No. 6, 11, 13, 14, 15, 16, 17, 18, 19, 21, 22	1.65 LPM He + 0.4 LPM He(80%)+ O_2(20%)	-	-	Open U-trap (No. 6).<br><br>*Safety note: watch the pressure at both pressure gauges (No. 8). If extreme pressures are observed, allow gas to escape the CHIPS by turning the flow towards the exit upstream of No. 11.*	Admitting the sample to the CHIPS.
**23**	Line through No. 11, 13, 14, 15, 16, 17, 18, 19, 21, 22	1.65 LPM He + 0.4 LPM He(80%)+ O_2(20%)	-	-	-	Additional time to ensure full sample collection.
**24**	Tail part (No. 18, 19, 21, 22)	-	Line through No. 11, 13, 14, 15, 16, 17	1.65 LPM He	Evacuate the tail part with vacuum pump (No. 10) followed by high-vacuum pump (No. 23) to < 1 mbar.<br><br>*Safety note: evacuation of No. 18, 19, 21, 22 is a safety measure to also remove potentially collected O_2.*	Cleaning No. 13, 14, and 16 from O_2 to reduce future CO_2 build-up. Evacuation No. 18, 19, 21, and 22 to remove potentially collected O_2.

**Time from start* (min)**	**CHIPS part in operation (numbers relate to elements of the CHIPS in Figure 2)**	**Flow in operational part**	**CHIPS part in recovery**	**Flow in recovery part**	**Additional action**	**Purpose of phase**
**28**	Line through No. 11, 15, 17, 18, 19, 21, 22	1.65 LPM He	-	-	First bypass No. 13, 14, and 16. Then fill the tail part (No. 18, 19, 21, 22) with He. Then let the flow exit at the tail end of the CHIPS.	Fill up the tail part to ambient pressure with He, followed by a continuous He flow exciting at the tail end of the CHIPS.
**31**	Line through No. 11, 15, 17, 18, 19, 21, 22	0.05 LPM He	-	-	Cool small CH_4-derived CO_2 collector (No. 19) with LN_2.	Preparing small CH_4-derived CO_2 collector (No. 19) for sub-transfer of collected CH_4-derived CO_2 in No. 18.
**36**	Line through No. 11, 15, 17, 18, 19, 21, 22	0.05 LPM He	-	-	Remove LN_2 from the large CH_4-derived CO_2 collector (No. 18) and warm to ambient temperature using warm air (e.g., a hair dryer).	Transfer of collected CH_4-derived CO_2 (in No. 18) to small CH_4-derived CO_2 collector (No. 19).
**56**	Tail part (No. 19, 21, 22)	-	Line through No. 11, 15, 17	0.05 LPM He	Evacuate the tail part with vacuum pump (No. 10) followed by high-vacuum pump (No. 23) to <0.01 mbar. Thereafter, remove the ethanol and dry ice slurry from the H_2O-scrubber (No. 17).	Evacuation of the tail part to remove any non-condensable gases. Cleaning No. 17 from collected H_2O.

Time from start* (min)	CHIPS part in operation (numbers relate to elements of the CHIPS in Figure 2)	Flow in operational part	CHIPS part in recovery	Flow in recovery part	Additional action	Purpose of phase
**58**	Tail part (No. 19)	-	Line through No. 11, 15, 17	0.05 LPM He	Isolate small CH_4-derived CO_2 collector (No. 19), remove LN_2, and warm to ambient temperature using warm air (e.g., a hair dryer).	Sublimation of collected CH_4-derived CO_2.
**61**	Tail part (No. 19)	-	Line through No. 11, 15, 17	0.05 LPM He	Put small CH_4-derived CO_2 collector (No. 19) in an ethanol and dry ice slurry.	Condense potentially co-trapped H_2O.
**64**	Tail part (No. 19, 21, 22)	-	Line through No. 11, 15, 17	0.05 LPM He	Evacuate manometric assembly and ampule (No. 21-22) with high-vacuum pump (No. 23) to <0.01 mbar. Then close the valve in between No. 21 and 22.	Evacuation of manometric assembly and ampule to remove any present gases.
**65**	Tail part (No. 19, 21)	-	Line through No. 11, 15, 17	0.05 LPM He	Put manometric assembly in LN_2 and allow cryo-transfer from No. 19 to No. 21.	Transfer of collected CH_4-derived CO_2 to manometric assembly (No. 21).

Time from start* (min)	CHIPS part in operation (numbers relate to elements of the CHIPS in Figure 2)	Flow in operational part	CHIPS part in recovery	Flow in recovery part	Additional action	Purpose of phase
**69**	Tail part (No. 21)	-	Line through No. 11, 15, 17	0.05 LPM He	Isolate manometric assembly (No. 21).<br><br>*Safety note: remove ethanol and dry ice slurry from No. 19 and clean No. 19 thereafter to have no ethanol close to the region of flame sealing.*	Isolation of collected CH_4-derived CO_2 in manometric assembly.
**71**	Tail part (No. 21, 22)	-	Line through No. 11, 15, 17	0.05 LPM He	Evacuate manometric assembly and ampule (No. 21-22) with high-vacuum pump (No. 23) to <0.01 mbar.<br>Re-isolate manometric assembly (No. 21), remove LN_2, and warm to ambient temperature using warm air (e.g., a hair dryer).	Evacuation of manometric assembly and ampule to remove any non-condensable gases.<br>Sublimation of collected CH_4-derived CO_2.
**73**	Tail part (No. 21)	-	Line through No. 11, 15, 17	0.05 LPM He	Put manometric assembly (No. 21) in water at room temperature and read the pressure with No. 20.	Manometric quantification of collected CH_4-derived CO_2.

Time from start* (min)	CHIPS part in operation (numbers relate to elements of the CHIPS in Figure 2)	Flow in operational part	CHIPS part in recovery	Flow in recovery part	Additional action	Purpose of phase
**74**	Tail part (No. 21, 22)	-	Line through No. 11, 15, 17	0.05 LPM He	Isolate and thereafter evacuate the ampule (No. 22) with high-vacuum pump (No. 23) to <0.01 mbar.	Prepare the ampule for cryo-transfer.
**75**	Tail part (No. 21, 22)	-	Line through No. 11, 15, 17	0.05 LPM He	Put the ampule in LN_2 and allow cryo-transfer from No. 21 to No. 22.	Transfer of collected CH_4-derived CO_2 to the ampule (No. 22).
**79**	Tail part (No. 21, 22)	-	Line through No. 11, 15, 17	0.05 LPM He	Evacuate manometric assembly and ampule (No. 21-22) with high-vacuum pump (No. 23) to <0.01 mbar. Thereafter, flame seal the ampule.<br><br>*Safety note: while sealing the ampule, monitor the pressure (at No. 20). Any observed leakage of air should be followed by immediate abortion of the process to omit condensing O_2.*	Evacuation of manometric assembly and ampule to remove any non-condensable gases. Production of the sample format, which can be sent off for radiocarbon analysis.

The timings are designed knowing that a 60 mL O_2 spike takes 6 min to travel through the full CHIPS until the tail of the O_2 peak is reduced to below the detection limit (<0.08% of the peak top height), as measured with a PreSens OXY-1 SMA-trace RS232-AO.

5. Details regarding the STRIPS and CHIPS method tests.

Table S7: Details regarding specific method tests of the STRIPS.

Method test	Procedure	Results
**CO_2 and H_20 removal in the gas-purifying canister**	The CO_2 and H_20 removal in the gas purifying canister setup was tested by continuous Cavity Ring-Down Spectrometer (CRDS)-monitoring (G2201-i Isotopic Analyzer, supplier: Picarro, Inc.) of both CO_2 and H_2O concentration for kegs which were filled with ~20 L tap water. The effectiveness of the H_2O and CO_2 removal was calculated based on the 5-minute average concentrations before leading the He flow through the gas purifying canister and the 3-3.5 h* average concentration while the flow was led through the gas purifying canister. During the CRDS monitoring, the flow rate through the STRIPS was 2.5 LPM**. The gas purifying canister was filled with 300 g indicating Drierite (10-20 mesh) and 100 g soda lime. Note that the gas purifying canister setup is similar to the setup that is used during STRIPS runs as described in the operational protocol.<br><br>**Note: This time is 1.2-1.4 times longer than that of a normal STRIPS run with a real sample.*<br>***This flow rate is 1.25 times higher than that used in a normal STRIPS run with a real sample.*	The H_2O and CO_2 concentrations measured by CRDS before circulation through the gas purifying canister were respectively 2.0±0.3% and 307±151 ppm (1σ, n=3).<br>The average H_2O and CO_2 concentrations measured by CRDS during the 3-3.5 h circulation through the gas purifying canister were respectively 0.015±0.006% and 1.2±1.9 ppm (1σ, n=3).<br>Thus, the average effectiveness of H_2O and CO_2 removal was 99.2±0.3% (1σ, n=3) and 99.6±0.6% (1σ, n=3), respectively.

Method test	Procedure	Results
**STRIPS yield**	The trapping and release yields of the STRIPS were tested by trapping CH_4 from ~20 L Swedish west-coast water*, spiked with 0.5 (n=1) or 2 mL (n=2) CH_4 followed by releasing the content of the produced and >4 weeks-stored U-trap in a verified CH_4-free He-filled STRIPS and keg. Apart from the keg preparation and spiking, all steps described in the STRIPS operational protocol were followed. The keg with ~20 L water was cleaned from ambient air using a He grade 5.0 flow of ~2 LPM for > 30 min. Spiking the keg with CH_4 was done after cleaning the keg and the STRIPS and after ~30 min of ~2 LPM circulation through the STRIPS (including the keg). After spiking, an equilibration period of 1h was executed, similar to the equilibration time in the operational protocol of the STRIPS.<br>The trapping and release yields were calculated as the ratio of the amount of CH_4 after release of CH_4 from the U-trap and equilibration into the CH_4-free He-filled STRIPS and keg and the amount of CH_4 in the STRIPS before trapping the CH_4 in the U-trap. The included parameters, each having their own uncertainty, for this calculation were thus the Henry constant for CH_4, the volume of water, the volume of the two kegs for CH_4 trapping and CH_4 release, the volume of the STRIPS, the pressure, temperature and CH_4 concentration in the STRIPS (including keg) before CH_4 trapping in the U-trap, and the pressure, temperature and CH_4 concentration in the STRIPS after CH_4 release and equilibration into the He-filled STRIPS and keg.<br>As a reference for maximum trapping and release yield, a similar experiment as above was executed by trapping CH_4 from seawater-free He-filled kegs spiked with 2 mL CH_4 (n=3) and <24 h of U-trap storage.<br>**The seawater used for these experiments was obtained from the Kristineberg Center at the Swedish west coast (salinity of ~33 PSU).*	The trapping and release yields (±1σ) for the kegs filled with ~20 L Swedish west-coast water were 99.7±2.4 (2 mL CH_4), 97.1±2.3 (2 mL CH_4), and 93.8±2.3 (0.5 mL CH_4).<br>The corresponding trapping efficiencies for these three runs were respectively >99.8%, >99.9%, and >99.4%.<br>The trapping and release yields (±1σ) for the kegs filled with He gas were 96.3±2.3 (2 mL CH_4), 98.3±2.2 (2 mL CH_4), and 98.5±2.1 (2 mL CH_4).<br>The corresponding trapping efficiencies for these three runs were respectively >99.7%, >99.7%, and >99.6%.

Table S8: Details regarding specific method tests of the CHIPS.

Method test	Procedure	Results
**CH_4 conversion in the main oven**	The conversion of CH_4 in the main oven (No. 16 in Figure 2) of the CHIPS was assessed by injecting, in 10 min repetition, CO_2 and CH_4 with a 500 µL sample loop attached at location No. 6 in Figure 2. The CO_2 and CH_4 concentrations were continuously CRDS-monitored, and by integrating the CO_2 peaks and calculating the ratio of CH_4-derived CO_2 peaks and CO_2-pulse CO_2 peaks, the CH_4 conversion yield was determined. The flowrates were as described in the CHIPS operational protocol (1.65 LPM He and 0.4 LPM O_2 and He (20:80)). Elements No. 11, 13, 14, and 15 (in Figure 2) were all bypassed during this experiment to not remove any CO_2 and purely assess CH_4 oxidation in the main oven.<br>In parallel, CO_2 was alternately bypassed and led through the main oven as a control measurement to determine whether any additional CO_2 was originating from it.	The CO_2 peak areas as measured by CRDS for CO_2 pulses bypassing the main oven, CO_2 pulses led through the main oven, and CH_4 pulses led through the main oven were 17048 ± 170 ppm·s (1σ, n=4), 17106 ± 176 ppm·s (1σ, n=4), and 16771 ± 94 ppm·s (1σ, n=4) respectively. Thus, the conversion yield of CH_4 in the main oven was 98.0±1.1% (1σ, n=4).<br>The ratio of CO_2 peak areas for CO_2 pulses led through and led past the main oven was 100.3±1.4% (1σ, n=4).
**CO conversion in the mild oven**	The oxidative removal of CO in the mild oven (~430 ^{o}C, No. 14 in Figure 2) of the CHIPS was assessed by continuous CRDS monitoring. For these conversion tests, 0.21 mL CO was injected through a syringe port downstream of the CH_4-scrubber (No. 4), and the gas flow was alternately guided through either the mild or main oven. By assuming that any conversion of CO results in CO_2 (i.e., oxidation) and CO is completely oxidized in the main oven, the conversion of CO was calculated as the ratio of the observed peak areas of CO_2 when guided through the mild and main oven. The flowrates were as described in the CHIPS operational protocol (1.65 LPM He and 0.4 LPM O_2 and He (20:80)) and elements No. 13 and 15 (in Figure 2) were not included during this experiment to not remove any CO-derived CO_2 and assess purely the conversion of CO in the mild oven.<br>To assess whether the mild oven at ~430 ^{o}C would affect CH_4, 0.23 mL CH_4 pulses, injected through a syringe port downstream of the CH_4-scrubber (No. 4), were alternately guided through and past the mild oven while continuously bypassing the main oven, and measuring the CH_4 peak areas by CRDS. The flow rates were as described in the CHIPS operational protocol (1.65 LPM He and 0.4 LPM O_2 and He (20:80)).	The CO_2 peak areas as measured by CRDS for CO pulses led through the mild oven and led through the main oven were 7749 ± 90 ppm·s (1σ, n=3) and 7639 ± 36 ppm·s (1σ, n=3) respectively. The conversion yield of CO in the mild oven was thus 101.4±1.3% (1σ, n=3).<br>The CH_4 peak areas as measured by CRDS for CH_4 pulses led through and led past the mild oven were 8230 ± 116 ppm·s (1σ, n=3) and 8406 ± 66 ppm·s (1σ, n=3) respectively. The amount of CH_4, which was reduced by the mild oven, was thus 2.1±1.6% (1σ, n=3).

Method test	Procedure	Results
**CO removal by the Sofnocat catalyst and mild oven**	The removal of CO by the combination of the mild oven (~430 ^{o}C, No. 14 in Figure 2) and the Sofnocat catalyst (No. 13) in the CHIPS was assessed by operating the CHIPS as normal, except for the injection of 0.24 mL CO through a syringe port upstream of No. 6 in Figure 2 (at the timing of opening a U-trap in a regular run). These runs were executed with and without the Sofnocat catalyst (No. 13 in Figure 2).<br>Continuous CRDS monitoring was used to assess whether the combination of the mild oven (No. 14) and Sofnocat catalyst (No. 13) affected the CH_4 presence. Pulses of 500 µL CH_4 were injected with a sample loop at location No. 6 in Figure 2 and alternately guided through and past the combination of these two elements. The resulting CH_4 peak areas were measured by CRDS. The flow rates for the CH_4 conservation tests were as described in the CHIPS operational protocol (1.65 LPM He and 0.4 LPM O_2 and He (20:80)), while all components of the CHIPS were included except the main oven.	The results of the CHIPS runs with CO injections can be found in Table S10.<br>The CH_4 peak areas as measured by CRDS for CH_4 pulses led through and led past the mild oven and Sofnocat catalyst were 18859 ± 94 ppm·s (1σ, n=3) and 18878 ± 160 ppm·s (1σ, n=3) respectively. The amount of CH_4 which was reduced by the mild oven was thus 0.1±1.0% (1σ, n=3).
**CHIPS blank**	The blank of the CHIPS was determined by operating the CHIPS as described in the operational protocol for the CHIPS while not opening the attached U-trap (No. 6 in Figure 2). Furthermore, different blank runs were executed to assess different components and setups of the CHIPS. The following variations were made: no mild oven nor Sofnocat catalyst, no Sofnocat catalyst but mild oven at 270 ^{o}C or 430 ^{o}C, and the combination of Sofnocat catalyst and mild oven at 430 ^{o}C (the combination as described in the operational protocol of the CHIPS). An overview of the setups of the blank runs can be found in Table S10.<br>A sub-part of the blank runs was collected in ampules and sealed. Two combined sets of such blanks were analyzed for radiocarbon content. The first set of blanks contained 11 blanks, all produced by operating the CHIPS without Sofnocat, 8 with the mild oven at 270 ^{o}C and 3 with the mild oven at 430 ^{o}C. The second set of blanks contained 17 blanks, all produced by operating the CHIPS with Sofnocat and mild oven at 430 ^{o}C, as described in the operational protocol of the CHIPS. Details regarding the subsets of the blank runs analyzed for radiocarbon content are shown in Table S10.	The carbon amounts for all blank runs of the CHIPS are shown in Table S10.<br>The AMS-determined $\Delta^{14}C$ (±1σ) of the first set of pooled blanks was -231±141‰. This blank $\Delta^{14}C$ value is used when the Sofnocat catalyst, for improved CO removal, is not included in the CHIPS. The AMS-determined $\Delta^{14}C$ (±1σ) of the second set of pooled blanks was -355±13‰.

Method test	Procedure	Results
**CO_2 removal**	To assess the removal of CO_2 in the CHIPS, CHIPS runs were executed with the admission of a pulse of 1 mL CO_2 through a syringe port downstream of the CH_4-scrubber (No. 4 in Figure 2) at the timing when a U-trap normally would be opened. The Sofnocat catalyst (No. 13) and mild oven (No. 14) were bypassed for these tests. Apart from these bypasses, the described operational protocol of the CHIPS was followed. These runs were compared to similarly executed CHIPS runs without CO_2 injection.	The results of the CHIPS runs with CO_2 injections can be found in Table S10.
**C_2H_6 removal**	To assess the removal of C_2H_6 in the CHIPS, CHIPS runs were executed with the admission of a pulse of 0.02 mL C_2H_6 through a syringe port downstream of the CH_4-scrubber (No. 4 in Figure 2) at the timing when a U-trap normally would be opened. The full operational protocol of the CHIPS was followed for these tests. The resulting values were compared to similar runs without the C_2H_6 injection.	The results of the CHIPS runs with C_2H_6 injections can be found in Table S10.

Table S9: Details regarding specific methods for testing the STRIPS and CHIPS combination.

Method part	Procedure
**Test water creation for the combined blank experiments of the STRIPS and the CHIPS**	A keg was filled with seawater to ~4.5-5.5 cm from the top, corresponding to a 100-400 mL headspace. The seawater type is detailed in Table S10. Thereafter, a headpiece similar to the custom-made headpiece (No. 2 in Figure 1) was put into the keg. The only difference in the headpiece was the addition of a sintered stainless-steel filter at the end of the longest tube of the headpiece to create small bubbles of He in the seawater during the test water creation. For two hours, 0.5 LPM He of grade 5.0 was admitted through this headpiece with filter into the seawater. Subsequently, the headpiece with the filter was removed and quickly replaced by a regular custom-made headpiece to simulate a cap swap for real samples. Thereafter a ~10 L headspace was created according to the procedure described in Table S3.
**Test water creation for the combined recovery tests for the STRIPS and the CHIPS**	The content of a keg, filled with ~20 L seawater, was cleaned with a ~2 LPM He flow through the customized headpiece (No. 2 in Figure 1) for 30 min. This keg with headpiece was thereafter directly connected to the STRIPS. This keg was subsequently taken through the regular STRIPS preparation steps (Table S3) and operational protocols (Table S4). The CH_4 (~0.5 mL CH_4 with $\Delta^{14}C\text{-}CH_4 < -997.3‰$) was added after cleaning the STRIPS, or after an additional ~30 min ~2 LPM circulation period through the STRIPS after cleaning the STRIPS (Table S11). Samples not spiked with ambient air (see details in Table S11) had this additional ~30 min circulation period before CH_4 injection, similar to the STRIPS yield experiments (Table S7). The seawater used for these experiments was obtained from the Kristineberg Center at the Swedish west coast (salinity of ~33 PSU).
**Calculating the yield of the combined operation of the STRIPS and the CHIPS**	The yield of the sequential operation of the STRIPS and the CHIPS was calculated as the ratio of the amount of CH_4-derived CO_2 as measured in the manometric assembly of the CHIPS (No. 21 in Figure 2) and the amount of CH_4 in the STRIPS before trapping CH_4 in the U-trap.<br>The included parameters, each with its own uncertainty, for this calculation were thus the Henry constant for CH_4, the volume of water, the volume of the keg, the volume of the STRIPS, the pressure, temperature, and CH_4 concentration in the STRIPS (including keg) before CH_4 trapping in the U-trap, the volume of the manometric assembly, the temperature and pressure of the gas in the manometric assembly, and the mass of the blank.

6. Experimental setups and results of individual tests: blanks of the CHIPS, blanks and yields of the STRIPS and the CHIPS.

Table S10: Specific experimental details and individual blanks of the CHIPS and the combination of the STRIPS and the CHIPS.

Included systems	Origin of water	Storage time U-trap (days)	Mild oven inclusion and temperature	Inclusion Sofnocat catalyst	Amount and material of injected gas	Blank amount (µg C)	CHIPS run No. of the day if not 1st	Analyzed by AMS
**CHIPS**			-	-		0.78		
**CHIPS**			-	-		0.66		
**CHIPS**			-	-		0.63	2nd	
**CHIPS**			-	-		0.72		
**CHIPS**			-	-		0.66	3rd	
**CHIPS**			-	-		1.76		
**CHIPS**			-	-		1.09		
**CHIPS**			-	-		0.98		
**CHIPS**			-	-		0.92	2nd	
**CHIPS & STRIPS**	Seawater at west-coast of Sweden*	9	-	-		1.01	3rd	
**CHIPS**			-	-	1 mL CO_2	1.09		
**CHIPS**			-	-	1 mL CO_2	0.72	2nd	
**CHIPS**			-	-	1 mL CO_2	0.66	3rd	
**CHIPS**			-	-	1 mL CO_2	0.63	4th	
**CHIPS**			Yes, 270 ^oC	-		0.98	2nd	
**CHIPS**			Yes, 270 ^oC	-		0.95		
**CHIPS**			Yes, 270 ^oC	-		0.86	2nd	
**CHIPS**			Yes, 270 ^oC	-		1.70		Set 1
**CHIPS**			Yes, 270 ^oC	-		1.15	2nd	Set 1
**CHIPS**			Yes, 270 ^oC	-		1.18		
**CHIPS**			Yes, 270 ^oC	-		0.86	2nd	Set 1
**CHIPS**			Yes, 270 ^oC	-		0.86		Set 1
**CHIPS**			Yes, 270 ^oC	-		1.01		
**CHIPS**			Yes, 270 ^oC	-		0.98		Set 1
**CHIPS**			Yes, 270 ^oC	-		1.21		Set 1
**CHIPS**			Yes, 270 ^oC	-		0.69		Set 1
**CHIPS**			Yes, 270 ^oC	-		0.63		Set 1
**CHIPS & STRIPS**	Seawater at west-coast of Sweden*	55	Yes, 270 ^oC	-		0.83	2nd	

Included systems	Origin of water	Storage time U-trap (days)	Mild oven inclusion and temperature	Inclusion Sofnocat catalyst	Amount and material of injected gas	Blank amount (µg C)	CHIPS run No. of the day if not 1st	Analyzed by AMS
**CHIPS & STRIPS**	Seawater at west-coast of Sweden*	23	Yes, 270 °C	-		0.78	4th	
**CHIPS & STRIPS**	Seawater at west-coast of Sweden*	218	Yes, 270 °C	-		0.92	2nd	
**CHIPS**			Yes, 430 °C	-		0.60		Set 1
**CHIPS**			Yes, 430 °C	-		0.63		Set 1
**CHIPS**			Yes, 430 °C	-		0.55	4th	Set 1
**CHIPS**			Yes, 430 °C	-		0.60		
**CHIPS**			Yes, 430 °C	-		0.72		
**CHIPS**			Yes, 430 °C	-	0.24 mL CO	1.96		
**CHIPS**			Yes, 430 °C	-	0.24 mL CO	1.38	2nd	
**CHIPS**			Yes, 430 °C	-	0.24 mL CO	1.06		
**CHIPS**			Yes, 430 °C	Yes		0.63		Set 2
**CHIPS**			Yes, 430 °C	Yes		0.75		Set 2
**CHIPS**			Yes, 430 °C	Yes		0.63		Set 2
**CHIPS**			Yes, 430 °C	Yes		0.66		Set 2
**CHIPS**			Yes, 430 °C	Yes		0.75		Set 2
**CHIPS**			Yes, 430 °C	Yes		0.78		Set 2
**CHIPS**			Yes, 430 °C	Yes		0.83		Set 2
**CHIPS**			Yes, 430 °C	Yes		0.72		Set 2
**CHIPS**			Yes, 430 °C	Yes		0.78		Set 2
**CHIPS**			Yes, 430 °C	Yes		0.66		Set 2
**CHIPS**			Yes, 430 °C	Yes		0.66		Set 2
**CHIPS**			Yes, 430 °C	Yes		0.66		Set 2
**CHIPS**			Yes, 430 °C	Yes		0.89		Set 2
**CHIPS**			Yes, 430 °C	Yes		0.69		Set 2
**CHIPS**			Yes, 430 °C	Yes		0.78		Set 2
**CHIPS**			Yes, 430 °C	Yes		0.66		Set 2
**CHIPS**			Yes, 430 °C	Yes		0.66		Set 2
**CHIPS**			Yes, 430 °C	Yes		0.52		
**CHIPS**			Yes, 430 °C	Yes		0.43	2nd	
**CHIPS**			Yes, 430 °C	Yes		0.60		
**CHIPS**			Yes, 430 °C	Yes		0.52		
**CHIPS**			Yes, 430 °C	Yes		0.43	3rd	
**STRIPS & CHIPS**	Seawater from Laptev Sea**	0	Yes, 430 °C	Yes		0.75	3rd	
**CHIPS**			Yes, 430 °C	Yes	0.24 mL CO	0.69		

Included systems	Origin of water	Storage time U-trap (days)	Mild oven inclusion and temperature	Inclusion Sofnocat catalyst	Amount and material of injected gas	Blank amount (µg C)	CHIPS run No. of the day if not 1st	Analyzed by AMS
**CHIPS**			Yes, 430 ^oC	Yes	0.24 mL CO	0.78		
**CHIPS**			Yes, 430 ^oC	Yes	0.24 mL CO	0.60	2nd	
**CHIPS**			Yes, 430 ^oC	Yes	0.24 mL CO	0.52	3rd	
**CHIPS**			Yes, 430 ^oC	Yes	0.24 mL CO	0.58		
**CHIPS**			Yes, 430 ^oC	Yes	0.02 mL C_2H_6	0.52		
**CHIPS**			Yes, 430 ^oC	Yes	0.02 mL C_2H_6	0.63		
**CHIPS**			Yes, 430 ^oC	Yes	0.02 mL C_2H_6	0.55		
**CHIPS**			Yes, 430 ^oC	Yes	0.02 mL C_2H_6	0.66		

**This seawater is obtained from the Kristineberg Center at the Swedish west coast (salinity of ~33 PSU).*

***This seawater is obtained from the Laptev Sea (76.9^{o}N, 127.8^{o}E, salinity of ~34 PSU).*

Table S11: Specific experimental details and yields for the combination of the STRIPS and the CHIPS.

Experiment sample type	Timing of CH_4 spiking	50 mL ambient air injected	U-trap storage time (days)	Mild oven T (^oC)	Inclusion Sofnocat catalyst	Blank-corrected sample size ± 1σ (μg C)	Yield ± 1σ (%)*
**CH_4-spiked seawater**	After 30 min of circulation through the STRIPS	No	97	Excluded	No	238.9±4.1	95.3±2.4
**CH_4-spiked seawater**	Directly after cleaning of the STRIPS	Yes	19	270	No	243.1±4.1	98.3±2.4
**CH_4-spiked seawater**	Directly after cleaning of the STRIPS	Yes	20	270	No	278.3±4.7	97.8±2.4
**CH_4-spiked seawater**	After 30 min of circulation through the STRIPS	No	323	430	No	241.4±4.1	95.1±2.4
**CH_4-spiked seawater**	Directly after cleaning of the STRIPS	Yes	218	430	No	325.8±5.5	94.6±2.3
**Baltic Sea sample**	-	-	13	270	No	35.4±0.6	100.2±2.9
**Baltic Sea sample**	-	-	14	270	No	598.3±38.5	97.5±6.6
**Inner Laptev Sea sample**	-	-	176	430	No	21.6±0.4	95.9±2.7
**Outer Laptev Sea sample**	-	-	174	430	No	418.2±7.0	97.3±2.7

**Note: the ratio of C amount measured at the AMS facility and our manometric quantified C amount (±1σ) was 1.09±0.06, which could imply that our estimated yield is slightly higher than our here reported values. As we do not have a verification of the C amount for every sample, we report all yields based on our manometric quantification for consistency.*

7. In-field sampling procedures.

Table S12: Procedures of filling sampling kegs.

Sampling region	Procedure
-	General approach for transferring sample water from Niskin/Go-Flo* flasks into a keg:<br><br>1. Rinse kegs prior to usage with a dilute (~1 M) HCl solution.<br>2. Sample seawater by triggering Niskin/Go-Flo flasks** at the specific water depth.<br>3. After retrieval of the Niskin/Go-Flo flasks, attach silicon tubing to the flask(s).<br>4. Rinse the attached silicon tubing with sample water and rinse the keg with 1-2 L of sample water.<br>5. Insert the rinsed tubing into the bottom of the rinsed keg and gently fill*** the keg with sample water by gravitational flow. For small Niskin flasks (< 30 L), insert rinsed tubing from parallel sampled flasks simultaneously. Inserting the tubing into the bottom of the keg is important to avoid excessive turbulence and degassing.<br>6. Overflow for a few seconds prior to the removal of the tubing.<br>7. Amend**** the sample with 125 mL 2kg/L $ZnCl_2$ solution.<br>8. Seal the keg with zero headspace using a polyoxymethylene (POM) lid and O-ring (Table S1).<br><br>Further note: transfer from other sample containers or direct<br><br>**The Go-Flo flask is a specialized and evolved form of the Niskin flask, manufactured by General Oceanics – Miami, FL, U.S.A. The used Go-Flo flask has a volume of 60 L.*<br>***Other sampling containers than Niskin or Go-Flo flasks or direct filling/pumping from (surface) water could be considered. For example, when sampling from lake/stream/river water. Care has to be taken to:*<br>- *Avoid contamination of other/previously sampled water by rinsing any connecting tubing or pump before filling the keg.*<br>- *Minimize turbulence and air-water contact time during filling.*<br>- *Spike the sampled water with $ZnCl_2$**** solution to avoid any microbial activity affecting the dissolved CH_4.*<br>*****During the filling of the kegs, the sampling flasks are displaced by ambient air, and also the keg is open to ambient air. As the air-sample area is small for the total volume of sample water and turbulence is minimized, it is assumed that the air-sample contact time (~10 minutes) is short enough to prevent any significant exchange of CH_4 between the gaseous and liquid phase. Sampled seawater in kegs showed, after 3-4 years of keg storage, no sign of lower concentration than those measured directly onboard.*<br>*****When the time between sampling and sample attachment to the STRIPS is less than a few hours, one does not need to add the preservative $ZnCl_2$.*

Sampling region	Procedure
**Landsort Deep, Baltic Sea**	The two sample kegs, filled with seawater sampled at 150 m and 430 m depth at Landsort Deep, were filled differently. Before usage, both kegs were cleaned with a dilute HCl solution. The water at each depth was sampled in a Go-Flo* flask.<br>Water sampled at 430 m depth was directly transferred from the Go-Flo flask to the bottom of the keg using silicon tubing. The silicon tubing connected to the Go-Flo flask was flushed with sampled seawater before filling the keg. During filling, the keg was allowed to overflow for ~10 s before capping it with a polyoxymethylene (POM) lid and O-ring (Table S1).<br>Water sampled at 150 m depth, was led into a pre-evacuated keg. The pre-evacuated keg with the customized headpiece (No. 2 in Figure 1) was prepared by evacuating and leak-checking the keg, filling the keg with He to ambient pressure, and then evacuating the keg to ~300 mbar. Silicon tubing attached to the Go-Flo flask was first flushed with sampled seawater before connecting the tubing to the customized headpiece. While the sampled seawater flowed through the customized headpiece into the keg, the pressure was monitored on the other tube of the headpiece with a pressure gauge. The flow was shut off when ambient pressure was reached. This resulted in a keg partly filled with seawater and partly having a He headspace.<br><br>**The Go-Flo flask is a specialized and evolved form of the Niskin flask, manufactured by General Oceanics – Miami, FL, U.S.A. The used Go-Flo flask has a volume of 60 L.*
**Laptev Sea, Arctic Ocean**	The kegs were pre-cleaned with a diluted (~1 M) HCl solution before usage. Seawater was sampled in four parallel sampled 10 L Niskin flasks. Attached silicon tubing to these flasks was in two pairs alternately fed into the kegs, where sample water was added to the keg only after rinsing the tubing. The kegs were first rinsed with 1-2 L water from the Niskin flasks and subsequently filled by feeding the silicon tubing to the bottom of the keg and allowing the kegs to fill slowly. Kegs were allowed to overflow for a few seconds prior to the removal of the tubing. Kegs were then dosed with 125 mL 2kg/L $ZnCl_2$ solution to kill any microbial activity that could potentially affect the present CH_4. Thereafter the kegs were quickly capped with a polyoxymethylene (POM) lid and O-ring (Table S1).

8. Isolated headspace creation system.

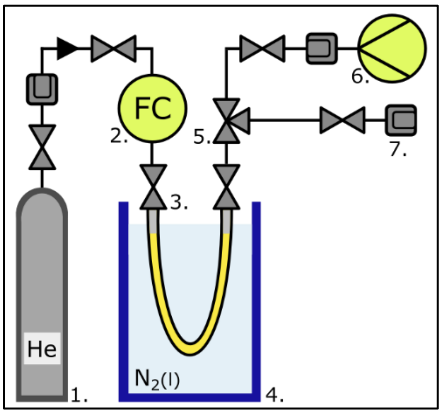

Figure S3: Flowchart of an isolated headspace creation system in the field.

1. Gas cylinder with He grade 5.0, 2. Flow controller (FC), 3. Toggle valve, 4. CH_4-scrubber cooled in LN_2-filled dewar flask, 5. 3-way valve, 6. Vacuum pump, 7. Quick-connect fitting. Note: during field applications, the gas cylinder, vacuum pump, and LN_2-filled dewar flask of the STIRPS can be used to minimize the materials to bring.

9. Comparison of existing methods and the STRIPS and the CHIPS.

Table S13: Comparison of existing methods for CH_4 extraction from ambient water and subsequent quantitative conversion to CO_2 for compound-specific radiocarbon analysis of CH_4 versus the STRIPS and the CHIPS.

	**STRIPS and CHIPS**	**Pohlman et al., 2000**	**Kessler & Reeburgh, 2005**	**Garnett et al., 2016**	**Sparrow & Kessler, 2017**	**Steinbach et al., 2021**
**Volume of water sampled**	30 L	Not reported.	114 L (smaller samples down to 19 L for water with higher CH_4 concentration)	4-9 L	20000 – 40000 L	40 – 80 L
**Location of CH_4 extraction from sample water**	In the field:<br>▪ Onboard.<br>▪ Any location to where LN_2 and He gas can be transported.	In the lab; one integrated system from dissolved CH_4 until graphitization.	In the field:<br>▪ Onboard<br>▪ Any location to where LN_2/dry ice/portable immersion cooler and He gas can be transported.	In the field.	In the field:<br>▪ Onboard.<br>▪ Any location facilitating the collection system and pressurized cylinders can be transported to.	In the field:<br>▪ Onboard.<br>▪ Any location to where LN_2 and He gas can be transported.
**Type and size of intermediate sample container**	Customized U-trap (Figure S1.3): ~35x12x5 cm	None.	Customized U-trap.	10 L foil gas sample bag.	1.7 L aluminum gas cylinder (240 L compressed gas)	Customized U-trap: ~35x12x5 cm
**Minimum sample size (C-CH_4)**	10 µg C	>55 µg C	22 µg C	500 µg C	200 µg C	Not reported. Smallest sample analyzed: 18 µg C
**Minimum CH_4 concentration in sample water**	40 nM*	Not reported.	15.9 nM	~4600 nM (~2100 nM when sequentially combining multiple samples)	2 nM	Not reported.

**Similar to other methods (Kessler & Reeburgh, 2005; Steinbach et al., 2021), we expect we can analyze samples with lower CH_4 concentrations using larger volumes of sample water, either by using a larger sample container or analyzing kegs in series. When increasing the total volume of water, we expect longer purging times to be necessary and possibly more Drierite and soda lime. While construction elements and sample type would be similar, we still recommend measuring the total blank for the combined setup to ensure the blank is also low for an altered setup.*

	STRIPS and CHIPS	Pohlman et al., 2000	Kessler & Reeburgh, 2005	Garnett et al., 2016	Sparrow & Kessler, 2017	Steinbach et al., 2021
**Estimated time of sampling**	<1/2 h	Not reported.	Not reported.	10-15 min (both sampling and CH_4 extraction)	~4 h (both sampling and CH_4 extraction)	<1/2 h
**Time for CH_4 extraction from sample water (part 1 of the method)**	~3 h (STRIPS)	~1/2 h (20 samples per day for both steps together)	~2 h			~2 h
**Time for CH_4 purification and conversion to CO_2 (part 2 of the method)**	~1.5 h (CHIPS)		>55 min	Not reported.	~3 h	Not reported.
**Ease of replicating measurements**	Easy:<br>▪ Fill multiple kegs with water sampled at the same location.<br>▪ Low and stable blank of the total method.	Unknown:<br>▪ Quick processing might facilitate replicates.<br>▪ Memory effect not tested.<br>▪ Unknown blank size and uncertainty.	Medium:<br>▪ Quick sampling, but no reports on storage effects/leakage of the carboys (gas-evacuated and only partly filled with sample water) before CH_4 extraction.	Easy:<br>▪ Quick sampling.<br>▪ Duplicate samples showed similar results.	Medium:<br>▪ Without replicating the build sampling and CH_4 extraction system, the long continuous sampling procedures impede simultaneous duplicate sampling.	Difficult:<br>▪ Duplicate samples are needed for CO_2 correction, which indirectly assumes that Δ^{14}C-CH_4 of duplicate samples is similar.
**Ease of replication of the method**	Likely possible:<br>▪ Construction parts, and preparation and operational protocols are described.	Not possible:<br>▪ Experimental details are missing.	Partly possible:<br>▪ The CH_4 extraction part can be replicated.<br>▪ Several experimental details are missing for (the operation of) the vacuum line.	Partly possible:<br>▪ Sampling and CH_4 extraction part can be replicated.<br>▪ Several experimental details are missing for the laboratory analyses.	Partly possible:<br>▪ The CH_4 extraction part can be replicated.<br>▪ Several experimental details are missing for (the operation of) the vacuum line.	Not possible:<br>▪ Several experimental details are missing.

	STRIPS and CHIPS	Pohlman et al., 2000	Kessler & Reeburgh, 2005	Garnett et al., 2016	Sparrow & Kessler, 2017	Steinbach et al., 2021
**Blank size (±1σ) of the total method***	0.67±0.12 µg C	Not reported.	6.3±4.7 µg C	Not reported. Furthermore, each sample needs to be corrected for 4-6 L ambient air contamination.	5.0±2.4 µg C	Not reported. Furthermore, each sample needs to be corrected for significant CO_2 presence.
**Δ^{14}C value of blank determined****	Yes	No	Yes	No	No	Partly (for the blank of part 2 of the method)
**Blank tested for removal efficacy of CO_2, C_2H_6, and CO****	Yes	No	No	No	No	No
**Removal step for CO****	Yes	No	Yes	No	Yes	Yes
**Removal step for C_2H_6****	Yes	Yes	Yes	No	Yes	Yes
**Stability of Δ^{14}C-CH_4 through all processing steps assessed****	Yes	No, but planned	No	Yes	No	No

Indicator of precision. Lower blank size and uncertainty indicate higher precision.

***Indicator of accuracy. "Yes" indicates higher accuracy.*

10. Global applicability of the STRIPS and the CHIPS for radiocarbon-based source apportionment of CH_4 in ambient water.

Table S14: Examples of global systems (ocean regimes and inland systems) where a CH_4 range has been reported to make it feasible to apply the STRIPS and the CHIPS for compound-specific radiocarbon analysis of CH_4.

Type	Sub-regions/types and example locations
**Ocean/ Sea**	Globally, emissions from oceans/seas are highest in **coastal and shallow regions** (Weber et al., 2019). In such regions, high enough CH_4 concentrations are found to apply the STRIPS and the CHIPS. Examples of such regions are:<br>• East Siberian Arctic Shelf (e.g., Shakhova et al., 2010)<br>• Chukchi Sea and Bering Strait (e.g., Kudo et al., 2022)<br>• Beaufort Sea (e.g., Lorenson et al., 2016)<br>• Continental Shelf of Spitsbergen (e.g., Damm et al., 2005)<br>• Baltic Sea (e.g., Ma et al., 2020)<br>• North Sea (e.g., Borges et al., 2018, 2019)<br>• Shallow Black Sea (e.g., Amouroux et al., 2002; Grilli et al., 2021; Malakhova et al., 2024)<br>• Shallow Caribbean Sea (e.g., Jones & Amador, 1993)<br>• Continental Shelf of Chile (e.g., Florez-Leiva et al., 2013)<br>• Continental Shelf of India (e.g., Shirodkar et al., 2018)<br>**Fjords**:<br>• West-Canada (e.g., Capelle et al., 2019)<br>• East-Canada (e.g., Li et al., 2021)<br>• Svalbard Archipelago (e.g., Damm et al., 2008)<br>**Estuaries**:<br>• Europe (e.g., Middelburg et al., 2002)<br>• U.S.A. (e.g., Barber et al., 1988)<br>• Canada (e.g., Y. Li et al., 2022)<br>• India (e.g., Araujo et al., 2018; Dutta et al., 2013)<br>• China (e.g., Ye et al., 2019; Zhou et al., 2009)<br>• Australia (e.g., Rosentreter et al., 2024)<br>Gas seepages, including mud volcanos and CH_4 hydrate decomposition (Etiope, 2015), and hydrothermal vents also cause elevated CH_4 concentrations in the water column. Examples of such locations where the STRIPS and CHIPS could be applied are:<br>**Seeps**:<br>• Cascadia Margin, Pacific Ocean (e.g., Heeschen et al., 2005)<br>• Atlantic Margin, Atlantic Ocean (e.g., Leonte et al., 2017, 2020)<br>• Svalbard Margin, Greenland Sea (e.g., Steinle et al., 2015)<br>• Norwegian Sea (e.g., Sauer et al., 2015)<br>• Black Sea (e.g., Kessler et al., 2006)<br>• Mediterranean Sea (e.g., Haese et al., 2003; Mastalerz et al., 2007)<br>• Gulf of Mexico (e.g., Leonte et al., 2018; Solomon et al., 2009)<br>• Caribbean Sea (e.g., Kessler et al., 2005, 2006)<br>• South China Sea (e.g., Di et al., 2019; N. Li et al., 2023)<br>**Hydrothermal vents**:<br>• Caribbean Sea (e.g., Bennett et al., 2013)<br>• Fuca Ridge, Pacific Ocean (e.g., de Angelis et al., 1993)<br>• Mid-Atlantic Ridge, Atlantic Ocean (e.g., Keir et al., 2009)

Type	Sub-regions/types and example locations
**Inland waters**	Globally, CH_4 emissions from inland waters are significant (Lauerwald et al., 2023; Zheng et al., 2022) and larger than coastal or oceanic emissions (Rosentreter et al., 2021). High CH_4 concentrations can be found in many inland water types, sufficient to apply the STRIPS and the CHIPS. Examples include:<br>**Lakes**:<br>• Siberia (e.g., Bussmann et al., 2021; Cabrol et al., 2020; Schmid et al., 2007; Shirokova et al., 2013)<br>• Alaska (e.g., Townsend-Small et al., 2017)<br>• Canada (e.g., Bogard et al., 2014; Mandryk et al., 2021)<br>• Tibetan Plateau (e.g., Mu et al., 2016; L. Wang et al., 2022)<br>• Greenland (e.g., Cadieux et al., 2022)<br>• Northern Europe (e.g., Bastviken et al., 2004; Juutinen et al., 2009; Peacock et al., 2023)<br>• Central Europe (e.g., Blees et al., 2015; Casper et al., 2003; Encinas Fernández et al., 2016; Günthel et al., 2019)<br>• Contiguous United States (e.g., Bastviken et al., 2004; Riera et al., 1999)<br>• Ecuador (e.g., Chiriboga & Borges, 2023)<br>• Chile (e.g., Gerardo-Nieto et al., 2017)<br>• Brazil (e.g., Marinho et al., 2009; Palma-Silva et al., 2013)<br>• Philippines (e.g., Mendoza-Pascual et al., 2021)<br>• Indonesia (e.g., Crowe et al., 2011)<br>• Japan (e.g., Murase et al., 2003)<br>• China (e.g., L. Li et al., 2018; H. Wang et al., 2021)<br>• Africa (e.g., Fazi et al., 2021; Morana et al., 2015, 2020; Zigah et al., 2015)<br>**Wetlands** (e.g., floodplains, swamps, marshes, bogs, peatland streams, ponds):<br>• Siberia (e.g., Langer et al., 2015; Rehder et al., 2021; Repo et al., 2007; Shirokova et al., 2013)<br>• Canada (e.g., Amaral & Knowles, 1994; Billett & Moore, 2008; Holgerson, 2015; Taillardat et al., 2024)<br>• U.S.A. (e.g., Barber et al., 1988; Holgerson, 2015; Hondula et al., 2021)<br>• Central and Northern Europe (e.g., Dinsmore et al., 2011; Evans et al., 2018; Holgerson, 2015; Hope et al., 2001; Natchimuthu et al., 2014)<br>• Brazil (e.g., Barbiero et al., 2018; Barbosa et al., 2020; Crill et al., 1988; Engle & Melack, 2000; Hamilton et al., 1995)<br>• Argentina (e.g., Münchberger et al., 2019)<br>• China (e.g., Ding et al., 2005)<br>• Thailand (e.g., Ueda et al., 2000)<br>• Australia (e.g., Jeffrey et al., 2019)<br>• Africa (e.g., Barthel et al., 2022; Lindholm et al., 2007)<br>**Rivers/tributaries**[*]:<br>• Siberia (e.g., Bussmann et al., 2021; Shakhova et al., 2007)<br>• Europe (e.g., Sieczko et al., 2016)<br>• South America (e.g., Barbosa et al., 2016; Chiriboga & Borges, 2023)<br>• East Asia (e.g., D. Wang et al., 2009; Zhang et al., 2021)<br>• Africa (e.g., Alberto V Borges et al., 2015; Bouillon et al., 2012; Teodoru et al., 2015; Upstill-Goddard et al., 2017)<br>**Glacial meltwater** (proglacial river/stream, proglacial lake, subglacial discharge):<br>• Antarctica (e.g., Wand et al., 2006)<br>• Tibetan Plateau (e.g., Yan et al., 2023)<br>• Svalbard (e.g., Kleber et al., 2024)<br>• Greenland (e.g., Dieser et al., 2014; Lamarche-Gagnon et al., 2019; Pain et al., 2021)<br>• Alaska (e.g., Konya et al., 2024)<br>• Canada (e.g., Sapper et al., 2023)

Type	Sub-regions/types and example locations
**Inland waters**	Next to natural inland waters with high CH_4 concentrations, there are human-affected inland waters like reservoirs (Rosentreter et al., 2021) for hydropower/water supply/irrigation and polluted/urban/aquaculture waters. Examples of such human-affected inland waters with CH_4 concentrations suitable for application of the STRIPS and the CHIPS are:<br>**Reservoirs**:<br>• U.S.A. (e.g., Beaulieu et al., 2014)<br>• Northern Europe (e.g., Huttunen et al., 2002)<br>• Central Europe (e.g., Diem et al., 2012)<br>• Brazil (e.g., de Araújo et al., 2024; Guérin et al., 2006; Kemenes et al., 2007, 2016; Quadra et al., 2020)<br>• French Guiana (e.g., Abril et al., 2005; Galy-Lacaux et al., 1997; Guérin et al., 2006)<br>• Chile (e.g., Gerardo-Nieto et al., 2017)<br>• China (e.g., Zhang et al., 2021)<br>• Laos (e.g., Chanudet et al., 2011)<br>• Africa (e.g., Teodoru et al., 2015)<br>**Polluted/urban/aquaculture waters:**<br>• Canada (e.g., Glaz et al., 2016)<br>• U.S.A. (e.g., Gorsky et al., 2024)<br>• Northern Europe (e.g., Audet et al., 2020; Peacock et al., 2019)<br>• Central Europe (e.g., Bauduin et al., 2024)<br>• Russia (e.g., Dzyuban, 2011)<br>• Brazil (e.g., Cotovicz et al., 2021)<br>• Mexico (e.g., Chuang et al., 2017)<br>• China (e.g., Hu et al., 2018; Kunpeng et al., 2019; B. Wang et al., 2021; R. Wang et al., 2020; X. Wang et al., 2022; Yang et al., 2019; Zhang et al., 2021)<br>• India (e.g., Pickard et al., 2021)<br>• Saudi Arabia (e.g., Orif et al., 2017)<br>• Australia (e.g., Bartosiewicz et al., 2021)

[*] A global database of dissolved methane in rivers can be found in Stanley et al. (2023).

11. References

Abril, G., Guérin, F., Richard, S., Delmas, R., Galy-Lacaux, C., Gosse, P., Tremblay, A., Varfalvy, L., Dos Santos, M. A., & Matvienko, B. (2005). Carbon dioxide and methane emissions and the carbon budget of a 10-year old tropical reservoir (Petit Saut, French Guiana). *Global Biogeochemical Cycles*, *19*(4). https://doi.org/10.1029/2005GB002457

Amaral, J. A., & Knowles, R. (1994). Methane Metabolism in a Temperate Swamp. *Applied and Environmental Microbiology*, *60*(11), 3945–3951. https://doi.org/10.1128/aem.60.11.3945-3951.1994

Amouroux, D., Roberts, G., Rapsomanikis, S., & Andreae, M. O. (2002). Biogenic Gas (CH4, N2O, DMS) Emission to the Atmosphere from Near-shore and Shelf Waters of the North-western Black Sea. *Estuarine, Coastal and Shelf Science*, *54*(3), 575–587. https://doi.org/10.1006/ecss.2000.0666

Araujo, J., Naqvi, S. W. A., Naik, H., & Naik, R. (2018). Biogeochemistry of methane in a tropical monsoonal estuarine system along the west coast of India. *Estuarine, Coastal and Shelf Science*, *207*, 435–443. https://doi.org/10.1016/j.ecss.2017.07.016

Audet, J., Carstensen, M. V., Hoffmann, C. C., Lavaux, L., Thiemer, K., & Davidson, T. A. (2020). Greenhouse gas emissions from urban ponds in Denmark. *Inland Waters*, *10*(3), 373–385. https://doi.org/10.1080/20442041.2020.1730680

Barber, T. R., Burke Jr., R. A., & Sackett, W. M. (1988). Diffusive flux of methane from warm wetlands. *Global Biogeochemical Cycles*, *2*(4), 411–425. https://doi.org/10.1029/GB002i004p00411

Barbiero, L., Siqueira Neto, M., Braz, R. R., Carmo, J. B. do, Rezende Filho, A. T., Mazzi, E., Fernandes, F. A., Damatto, S. R., & Camargo, P. B. de. (2018). Biogeochemical diversity, O2-supersaturation and hot moments of GHG emissions from shallow alkaline lakes in the Pantanal of Nhecolândia, Brazil. *Science of The Total Environment*, *619–620*, 1420–1430. https://doi.org/10.1016/j.scitotenv.2017.11.197

Barbosa, P. M., Melack, J. M., Amaral, J. H. F., MacIntyre, S., Kasper, D., Cortés, A., Farjalla, V. F., & Forsberg, B. R. (2020). Dissolved methane concentrations and fluxes to the atmosphere from a tropical floodplain lake. *Biogeochemistry*, *148*(2), 129–151. https://doi.org/10.1007/s10533-020-00650-1

Barbosa, P. M., Melack, J. M., Farjalla, V. F., Amaral, J. H. F., Scofield, V., & Forsberg, B. R. (2016). Diffusive methane fluxes from Negro, Solimões and Madeira rivers and fringing lakes in the Amazon basin. *Limnology and Oceanography*, *61*(S1), S221–S237. https://doi.org/10.1002/lno.10358

Barthel, M., Bauters, M., Baumgartner, S., Drake, T. W., Bey, N. M., Bush, G., Boeckx, P., Botefa, C. I., Dériaz, N., Ekamba, G. L., Gallarotti, N., Mbayu, F. M., Mugula, J. K., Makelele, I. A., Mbongo, C. E., Mohn, J., Mandea, J. Z., Mpambi, D. M., Ntaboba, L. C., … Six, J. (2022). Low N2O and variable CH4 fluxes from tropical forest soils of the Congo Basin. *Nature Communications*, *13*(1), 330. https://doi.org/10.1038/s41467-022-27978-6

Bartosiewicz, M., Coggins, L. X., Glaz, P., Cortés, A., Bourget, S., Reichwaldt, E. S., MacIntyre, S., Ghadouani, A., & Laurion, I. (2021). Integrated approach towards quantifying carbon dioxide and methane release from waste stabilization ponds. *Water Research*, *202*, 117389. https://doi.org/10.1016/j.watres.2021.117389

Bastviken, D., Cole, J., Pace, M., & Tranvik, L. (2004). Methane emissions from lakes: Dependence of lake characteristics, two regional assessments, and a global estimate. *Global Biogeochemical Cycles*, *18*(4). https://doi.org/10.1029/2004GB002238

Bauduin, T., Gypens, N., & Borges, A. V. (2024). Seasonal and spatial variations of greenhouse gas (CO2, CH4 and N2O) emissions from urban ponds in Brussels. *Water Research*, *253*, 121257. https://doi.org/10.1016/j.watres.2024.121257

Beaulieu, J. J., Smolenski, R. L., Nietch, C. T., Townsend-Small, A., & Elovitz, M. S. (2014). High Methane Emissions from a Midlatitude Reservoir Draining an Agricultural Watershed. *Environmental Science & Technology*, *48*(19), 11100–11108. https://doi.org/10.1021/es501871g

Bennett, S. A., Coleman, M., Huber, J. A., Reddington, E., Kinsey, J. C., McIntyre, C., Seewald, J. S., & German, C. R. (2013). Trophic regions of a hydrothermal plume dispersing away from an ultramafic-hosted vent-system: Von Damm vent-site, Mid-Cayman Rise. *Geochemistry, Geophysics, Geosystems*, *14*(2), 317–327. https://doi.org/10.1002/ggge.20063

Billett, M. F., & Moore, T. R. (2008). Supersaturation and evasion of CO2 and CH4 in surface waters at Mer Bleue peatland, Canada. *Hydrological Processes*, *22*(12), 2044–2054. https://doi.org/10.1002/hyp.6805

Blees, J., Niemann, H., Erne, M., Zopfi, J., Schubert, C. J., & Lehmann, M. F. (2015). Spatial variations in surface water methane super-saturation and emission in Lake Lugano, southern Switzerland. *Aquatic Sciences*, *77*(4), 535–545. https://doi.org/10.1007/s00027-015-0401-z

Bogard, M. J., del Giorgio, P. A., Boutet, L., Chaves, M. C. G., Prairie, Y. T., Merante, A., & Derry, A. M. (2014). Oxic water column methanogenesis as a major component of aquatic CH4 fluxes. *Nature Communications*, *5*(1), 5350. https://doi.org/10.1038/ncomms6350

Borges, A. V., Royer, C., Martin, J. L., Champenois, W., & Gypens, N. (2019). Response of marine methane dissolved concentrations and emissions in the Southern North Sea to the European 2018 heatwave. *Continental Shelf Research*, *190*, 104004. https://doi.org/10.1016/j.csr.2019.104004

Borges, A. V., Speeckaert, G., Champenois, W., Scranton, M. I., & Gypens, N. (2018). Productivity and Temperature as Drivers of Seasonal and Spatial Variations of Dissolved Methane in the Southern Bight of the North Sea. *Ecosystems*, *21*(4), 583–599. https://doi.org/10.1007/s10021-017-0171-7

Borges, Alberto V, Darchambeau, F., Teodoru, C. R., Marwick, T. R., Tamooh, F., Geeraert, N., Omengo, F. O., Guérin, F., Lambert, T., Morana, C., Okuku, E., & Bouillon, S. (2015). Globally significant greenhouse-gas emissions from African inland waters. *Nature Geoscience*, *8*(8), 637–642. https://doi.org/10.1038/ngeo2486

Bouillon, S., Yambélé, A., Spencer, R. G. M., Gillikin, D. P., Hernes, P. J., Six, J., Merckx, R., & Borges, A. V. (2012). Organic matter sources, fluxes and greenhouse gas exchange in the Oubangui River (Congo River basin). *Biogeosciences*, *9*(6), 2045–2062. https://doi.org/10.5194/bg-9-2045-2012

Bussmann, I., Fedorova, I., Juhls, B., Overduin, P. P., & Winkel, M. (2021). Methane dynamics in three different Siberian water bodies under winter and summer conditions. *Biogeosciences*, *18*(6), 2047–2061. https://doi.org/10.5194/bg-18-2047-2021

Cabrol, L., Thalasso, F., Gandois, L., Sepulveda-Jauregui, A., Martinez-Cruz, K., Teisserenc, R., Tananaev, N., Tveit, A., Svenning, M. M., & Barret, M. (2020). Anaerobic oxidation of methane

and associated microbiome in anoxic water of Northwestern Siberian lakes. *Science of The Total Environment*, *736*, 139588. https://doi.org/10.1016/j.scitotenv.2020.139588

Cadieux, S. B., Schütte, U. M. E., Hemmerich, C., Powers, S., & White, J. R. (2022). Exploring methane cycling in an arctic lake in Kangerlussuaq Greenland using stable isotopes and 16S rRNA gene sequencing. *Frontiers in Environmental Science*, *10*. https://www.frontiersin.org/journals/environmental-science/articles/10.3389/fenvs.2022.884133

Capelle, D. W., Hallam, S. J., & Tortell, P. D. (2019). Time-series CH4 measurements from Saanich Inlet, BC, a seasonally anoxic fjord. *Marine Chemistry*, *215*, 103664. https://doi.org/10.1016/j.marchem.2019.103664

Casper, P., Chim Chan, O., Furtado, A. L. S., & Adams, D. D. (2003). Methane in an acidic bog lake: The influence of peat in the catchment on the biogeochemistry of methane. *Aquatic Sciences*, *65*(1), 36–46. https://doi.org/10.1007/s000270300003

Chanudet, V., Descloux, S., Harby, A., Sundt, H., Hansen, B. H., Brakstad, O., Serça, D., & Guerin, F. (2011). Gross CO2 and CH4 emissions from the Nam Ngum and Nam Leuk sub-tropical reservoirs in Lao PDR. *Science of The Total Environment*, *409*(24), 5382–5391. https://doi.org/10.1016/j.scitotenv.2011.09.018

Chiriboga, G., & Borges, A. V. (2023). Andean headwater and piedmont streams are hot spots of carbon dioxide and methane emissions in the Amazon basin. *Communications Earth & Environment*, *4*(1), 76. https://doi.org/10.1038/s43247-023-00745-1

Chuang, P.-C., Young, M. B., Dale, A. W., Miller, L. G., Herrera-Silveira, J. A., & Paytan, A. (2017). Methane fluxes from tropical coastal lagoons surrounded by mangroves, Yucatán, Mexico. *Journal of Geophysical Research: Biogeosciences*, *122*(5), 1156–1174. https://doi.org/10.1002/2017JG003761

Cotovicz, L. C., Ribeiro, R. P., Régis, C. R., Bernardes, M., Sobrinho, R., Vidal, L. O., Tremmel, D., Knoppers, B. A., & Abril, G. (2021). Greenhouse gas emissions (CO2 and CH4) and inorganic carbon behavior in an urban highly polluted tropical coastal lagoon (SE, Brazil). *Environmental Science and Pollution Research*, *28*(28), 38173–38192. https://doi.org/10.1007/s11356-021-13362-2

Crill, P. M., Bartlett, K. B., Wilson, J. O., Sebacher, D. I., Harriss, R. C., Melack, J. M., MacIntyre, S., Lesack, L., & Smith-Morrill, L. (1988). Tropospheric methane from an Amazonian floodplain lake. *Journal of Geophysical Research: Atmospheres*, *93*(D2), 1564–1570. https://doi.org/10.1029/JD093iD02p01564

Crowe, S. A., Katsev, S., Leslie, K., Sturm, A., Magen, C., Nomosatyro, S., Pack, M. A., Kessler, J. D., Reeburgh, W. S., Roberts, J. A., Gonzalez, L., Douglas Haffner, G., Mucci, A., Sundby, B., & Fowle, D. A. (2011). The methane cycle in ferruginous Lake Matano. *Geobiology*, *9*(1), 61–78. https://doi.org/10.1111/j.1472-4669.2010.00257.x

Damm, E., Kiene, R. P., Schwarz, J., Falck, E., & Dieckmann, G. (2008). Methane cycling in Arctic shelf water and its relationship with phytoplankton biomass and DMSP. *Marine Chemistry*, *109*(1), 45–59. https://doi.org/10.1016/j.marchem.2007.12.003

Damm, E., Mackensen, A., Budéus, G., Faber, E., & Hanfland, C. (2005). Pathways of methane in seawater: Plume spreading in an Arctic shelf environment (SW-Spitsbergen). *Continental Shelf Research*, *25*(12), 1453–1472. https://doi.org/10.1016/j.csr.2005.03.003

de Angelis, M. A., Lilley, M. D., & Baross, J. A. (1993). Methane oxidation in deep-sea hydrothermal

plumes of the endeavour segment of the Juan de Fuca Ridge. *Deep Sea Research Part I: Oceanographic Research Papers*, *40*(6), 1169–1186. https://doi.org/10.1016/0967-0637(93)90132-M

de Araújo, K. R., Sawakuchi, H. O., Bertassoli, D. J., Bastviken, D., Pereira, T. S., & Sawakuchi, A. O. (2024). Operational effects on aquatic carbon dioxide and methane emissions from the Belo Monte hydropower plant in the Xingu River, eastern Amazonia. *Science of The Total Environment*, *946*, 174100. https://doi.org/10.1016/j.scitotenv.2024.174100

Di, P., Feng, D., & Chen, D. (2019). The Distribution of Dissolved Methane and Its Air-Sea Flux in the Plume of a Seep Field, Lingtou Promontory, South China Sea. *Geofluids*, *2019*(1), 3240697. https://doi.org/10.1155/2019/3240697

Diem, T., Koch, S., Schwarzenbach, S., Wehrli, B., & Schubert, C. J. (2012). Greenhouse gas emissions (CO2, CH4, and N2O) from several perialpine and alpine hydropower reservoirs by diffusion and loss in turbines. *Aquatic Sciences*, *74*(3), 619–635. https://doi.org/10.1007/s00027-012-0256-5

Dieser, M., Broemsen, E. L. J. E., Cameron, K. A., King, G. M., Achberger, A., Choquette, K., Hagedorn, B., Sletten, R., Junge, K., & Christner, B. C. (2014). Molecular and biogeochemical evidence for methane cycling beneath the western margin of the Greenland Ice Sheet. *The ISME Journal*, *8*(11), 2305–2316. https://doi.org/10.1038/ismej.2014.59

Ding, W., Cai, Z., & Tsuruta, H. (2005). Factors affecting seasonal variation of methane concentration in water in a freshwater marsh vegetated with Carex lasiocarpa. *Biology and Fertility of Soils*, *41*, 1–8. https://doi.org/10.1007/s00374-004-0812-9

Dinsmore, K. J., Smart, R. P., Billett, M. F., Holden, J., Baird, A. J., & Chapman, P. J. (2011). Greenhouse gas losses from peatland pipes: A major pathway for loss to the atmosphere? *Journal of Geophysical Research: Biogeosciences*, *116*(G3). https://doi.org/10.1029/2011JG001646

Dutta, M. K., Chowdhury, C., Jana, T. K., & Mukhopadhyay, S. K. (2013). Dynamics and exchange fluxes of methane in the estuarine mangrove environment of the Sundarbans, NE coast of India. *Atmospheric Environment*, *77*, 631–639. https://doi.org/10.1016/j.atmosenv.2013.05.050

Dzyuban, A. N. (2011). Methane and its transformation processes in water of some tributaries of the Rybinsk Reservoir. *Water Resources*, *38*(5), 615–620. https://doi.org/10.1134/S0097807811050046

Encinas Fernández, J., Peeters, F., & Hofmann, H. (2016). On the methane paradox: Transport from shallow water zones rather than in situ methanogenesis is the major source of CH4 in the open surface water of lakes. *Journal of Geophysical Research: Biogeosciences*, *121*(10), 2717–2726. https://doi.org/10.1002/2016JG003586

Engle, D., & Melack, J. M. (2000). Methane emissions from an Amazon floodplain lake: Enhanced release during episodic mixing and during falling water. *Biogeochemistry*, *51*(1), 71–90. https://doi.org/10.1023/A:1006389124823

Etiope, G. (2015). Natural Gas Seepage: The Earth’s Hydrocarbon Degassing. In *Natural Gas Seepage: The Earth’s Hydrocarbon Degassing*. https://doi.org/10.1007/978-3-319-14601-0

Evans, C. D., Peacock, M., Green, S. M., Holden, J., Chapman, P. J., Lebron, I., Callaghan, N., Grayson, R., & Baird, A. J. (2018). The impact of ditch blocking on fluvial carbon export from a UK blanket bog. *Hydrological Processes*, *32*(13), 2141–2154. https://doi.org/10.1002/hyp.13158

Fazi, S., Amalfitano, S., Venturi, S., Pacini, N., Vazquez, E., Olaka, L. A., Tassi, F., Crognale, S.,

Herzsprung, P., Lechtenfeld, O. J., Cabassi, J., Capecchiacci, F., Rossetti, S., Yakimov, M. M., Vaselli, O., Harper, D. M., & Butturini, A. (2021). High concentrations of dissolved biogenic methane associated with cyanobacterial blooms in East African lake surface water. *Communications Biology*, *4*(1), 845. https://doi.org/10.1038/s42003-021-02365-x

Florez-Leiva, L., Damm, E., & Farías, L. (2013). Methane production induced by dimethylsulfide in surface water of an upwelling ecosystem. *Progress in Oceanography*, *112–113*, 38–48. https://doi.org/10.1016/j.pocean.2013.03.005

Galy-Lacaux, C., Delmas, R., Jambert, C., Dumestre, J.-F., Labroue, L., Richard, S., & Gosse, P. (1997). Gaseous emissions and oxygen consumption in hydroelectric dams: A case study in French Guyana. *Global Biogeochemical Cycles*, *11*(4), 471–483. https://doi.org/10.1029/97GB01625

Garnett, M. H., Gulliver, P., & Billett, M. F. (2016). A rapid method to collect methane from peatland streams for radiocarbon analysis. *Ecohydrology*, *9*(1), 113–121. https://doi.org/10.1002/eco.1617

Gerardo-Nieto, O., Astorga-España, M. S., Mansilla, A., & Thalasso, F. (2017). Initial report on methane and carbon dioxide emission dynamics from sub-Antarctic freshwater ecosystems: A seasonal study of a lake and a reservoir. *Science of The Total Environment*, *593–594*, 144–154. https://doi.org/10.1016/j.scitotenv.2017.02.144

Glaz, P., Bartosiewicz, M., Laurion, I., Reichwaldt, E. S., Maranger, R., & Ghadouani, A. (2016). Greenhouse gas emissions from waste stabilisation ponds in Western Australia and Quebec (Canada). *Water Research*, *101*, 64–74. https://doi.org/10.1016/j.watres.2016.05.060

Gorsky, A. L., Dugan, H. A., Wilkinson, G. M., & Stanley, E. H. (2024). Under-Ice Oxygen Depletion and Greenhouse Gas Supersaturation in North Temperate Urban Ponds. *Journal of Geophysical Research: Biogeosciences*, *129*(6), e2024JG008120. https://doi.org/10.1029/2024JG008120

Grilli, R., Birot, D., Schumacher, M., Paris, J.-D., Blouzon, C., Donval, J. P., Guyader, V., Leau, H., Giunta, T., Delmotte, M., Radulescu, V., Balan, S., Greinert, J., & Ruffine, L. (2021). Inter-Comparison of the Spatial Distribution of Methane in the Water Column From Seafloor Emissions at Two Sites in the Western Black Sea Using a Multi-Technique Approach. *Frontiers in Earth Science*, *9*. https://www.frontiersin.org/journals/earth-science/articles/10.3389/feart.2021.626372

Guérin, F., Abril, G., Richard, S., Burban, B., Reynouard, C., Seyler, P., & Delmas, R. (2006). Methane and carbon dioxide emissions from tropical reservoirs: Significance of downstream rivers. *Geophysical Research Letters*, *33*(21). https://doi.org/10.1029/2006GL027929

Günthel, M., Donis, D., Kirillin, G., Ionescu, D., Bizic, M., McGinnis, D. F., Grossart, H.-P., & Tang, K. W. (2019). Contribution of oxic methane production to surface methane emission in lakes and its global importance. *Nature Communications*, *10*(1), 5497. https://doi.org/10.1038/s41467-019-13320-0

Haese, R. R., Meile, C., Van Cappellen, P., & De Lange, G. J. (2003). Carbon geochemistry of cold seeps: Methane fluxes and transformation in sediments from Kazan mud volcano, eastern Mediterranean Sea. *Earth and Planetary Science Letters*, *212*(3), 361–375. https://doi.org/10.1016/S0012-821X(03)00226-7

Hamilton, S. K., Sippel, S. J., & Melack, J. M. (1995). Oxygen depletion and carbon dioxide and methane production in waters of the Pantanal wetland of Brazil. *Biogeochemistry*, *30*(2), 115–141. https://doi.org/10.1007/BF00002727

Heeschen, K. U., Collier, R. W., de Angelis, M. A., Suess, E., Rehder, G., Linke, P., & Klinkhammer, G.

P. (2005). Methane sources, distributions, and fluxes from cold vent sites at Hydrate Ridge, Cascadia Margin. *Global Biogeochemical Cycles*, *19*(2). https://doi.org/10.1029/2004GB002266

Holgerson, M. A. (2015). Drivers of carbon dioxide and methane supersaturation in small, temporary ponds. *Biogeochemistry*, *124*(1), 305–318. https://doi.org/10.1007/s10533-015-0099-y

Hondula, K., Jones, C., & Palmer, M. (2021). Effects of seasonal inundation on methane fluxes from forested freshwater wetlands. *Environmental Research Letters*, *16*. https://doi.org/10.1088/1748-9326/ac1193

Hope, D., Palmer, S., Billett, M., & Julián, J. M. (2001). Carbon Dioxide and Methane Evasion from a Temperate Peatland Stream. *Limnology and Oceanography*, *46*, 847–857. https://doi.org/10.4319/lo.2001.46.4.0847

Hu, B., Wang, D., Zhou, J., Meng, W., Li, C., Sun, Z., Guo, X., & Wang, Z. (2018). Greenhouse gases emission from the sewage draining rivers. *Science of The Total Environment*, *612*, 1454–1462. https://doi.org/10.1016/j.scitotenv.2017.08.055

Huttunen, J. T., Väisänen, T. S., Hellsten, S. K., Heikkinen, M., Nykänen, H., Jungner, H., Niskanen, A., Virtanen, M. O., Lindqvist, O. V, Nenonen, O. S., & Martikainen, P. J. (2002). Fluxes of CH4, CO2, and N2O in hydroelectric reservoirs Lokka and Porttipahta in the northern boreal zone in Finland. *Global Biogeochemical Cycles*, *16*(1), 3–17. https://doi.org/10.1029/2000GB001316

Jeffrey, L. C., Maher, D. T., Johnston, S. G., Kelaher, B. P., Steven, A., & Tait, D. R. (2019). Wetland methane emissions dominated by plant-mediated fluxes: Contrasting emissions pathways and seasons within a shallow freshwater subtropical wetland. *Limnology and Oceanography*, *64*(5), 1895–1912. https://doi.org/10.1002/lno.11158

Jones, R. D., & Amador, J. A. (1993). Methane and carbon monoxide production, oxidation, and turnover times in the Caribbean Sea as influenced by the Orinoco River. *Journal of Geophysical Research: Oceans*, *98*(C2), 2353–2359. https://doi.org/10.1029/92JC02769

Juutinen, S., Rantakari, M., Kortelainen, P., Huttunen, J. T., Larmola, T., Alm, J., Silvola, J., & Martikainen, P. J. (2009). Methane dynamics in different boreal lake types. *Biogeosciences*, *6*(2), 209–223. https://doi.org/10.5194/bg-6-209-2009

Keir, R. S., Schmale, O., Seifert, R., & Sültenfuß, J. (2009). Isotope fractionation and mixing in methane plumes from the Logatchev hydrothermal field. *Geochemistry, Geophysics, Geosystems*, *10*(5). https://doi.org/10.1029/2009GC002403

Kemenes, A., Forsberg, B. R., & Melack, J. M. (2007). Methane release below a tropical hydroelectric dam. *Geophysical Research Letters*, *34*(12). https://doi.org/10.1029/2007GL029479

Kemenes, A., Forsberg, B. R., & Melack, J. M. (2016). Downstream emissions of CH4 and CO2 from hydroelectric reservoirs (Tucuruí, Samuel, and Curuá-Una) in the Amazon basin . *Inland Waters*, *6*(3), 295–302. https://doi.org/10.1080/IW-6.3.980

Kessler, J. D., & Reeburgh, W. S. (2005). Preparation of natural methane samples for stable isotope and radiocarbon analysis. *Limnology and Oceanography: Methods*, *3*(9), 408–418. https://doi.org/10.4319/lom.2005.3.408

Kessler, J. D., Reeburgh, W. S., Southon, J., & Varela, R. (2005). Fossil methane source dominates Cariaco Basin water column methane geochemistry. *Geophysical Research Letters*, *32*(12). https://doi.org/10.1029/2005GL022984

Kessler, J. D., Reeburgh, W. S., & Tyler, S. C. (2006). Controls on methane concentration and stable isotope (δ2H-CH4 and δ13C-CH4) distributions in the water columns of the Black Sea and

Cariaco Basin. *Global Biogeochemical Cycles*, *20*(4). https://doi.org/10.1029/2005GB002571

Kleber, G. E., Magerl, L., Turchyn, A. V, Trimmer, M., Zhu, Y., & Hodson, A. (2024). Proglacial methane emissions driven by meltwater and groundwater flushing in a high Arctic glacial catchment. *EGUsphere*, *2024*, 1–23. https://doi.org/10.5194/egusphere-2024-1273

Konya, K., Sueyoshi, T., Iwahana, G., Morishita, T., Uetake, J., & Wakita, M. (2024). CH4 emissions from runoff water of Alaskan mountain glaciers. *Scientific Reports*, *14*(1), 10558. https://doi.org/10.1038/s41598-024-56608-y

Kudo, K., Toyoda, S., Yamada, K., Yoshida, N., Sasano, D., Kosugi, N., Murata, A., Uchida, H., & Nishino, S. (2022). Source analysis of dissolved methane in Chukchi Sea and Bering Strait during summer–autumn of 2012 and 2013. *Marine Chemistry*, *243*, 104119. https://doi.org/10.1016/j.marchem.2022.104119

Kunpeng, Z., Nan, Z., Xuemei, X., Lingxi, Z., & Juying, W. (2019). Bubble-mediated methane release from polluted Dalian Bay in China in summer, 2016. *Continental Shelf Research*, *185*, 51–56. https://doi.org/10.1016/j.csr.2018.11.009

Lamarche-Gagnon, G., Wadham, J. L., Sherwood Lollar, B., Arndt, S., Fietzek, P., Beaton, A. D., Tedstone, A. J., Telling, J., Bagshaw, E. A., Hawkings, J. R., Kohler, T. J., Zarsky, J. D., Mowlem, M. C., Anesio, A. M., & Stibal, M. (2019). Greenland melt drives continuous export of methane from the ice-sheet bed. *Nature*, *565*(7737), 73–77. https://doi.org/10.1038/s41586-018-0800-0

Langer, M., Westermann, S., Walter Anthony, K., Wischnewski, K., & Boike, J. (2015). Frozen ponds: production and storage of methane during the Arctic winter in a lowland tundra landscape in northern Siberia, Lena River delta. *Biogeosciences*, *12*(4), 977–990. https://doi.org/10.5194/bg-12-977-2015

Lauerwald, R., Allen, G. H., Deemer, B. R., Liu, S., Maavara, T., Raymond, P., Alcott, L., Bastviken, D., Hastie, A., Holgerson, M. A., Johnson, M. S., Lehner, B., Lin, P., Marzadri, A., Ran, L., Tian, H., Yang, X., Yao, Y., & Regnier, P. (2023). Inland Water Greenhouse Gas Budgets for RECCAP2: 1. State-Of-The-Art of Global Scale Assessments. *Global Biogeochemical Cycles*, *37*(5), e2022GB007657. https://doi.org/10.1029/2022GB007657

Leonte, M., Kessler, J. D., Kellermann, M. Y., Arrington, E. C., Valentine, D. L., & Sylva, S. P. (2017). Rapid rates of aerobic methane oxidation at the feather edge of gas hydrate stability in the waters of Hudson Canyon, US Atlantic Margin. *Geochimica et Cosmochimica Acta*, *204*, 375–387. https://doi.org/10.1016/j.gca.2017.01.009

Leonte, M., Ruppel, C. D., Ruiz-Angulo, A., & Kessler, J. D. (2020). Surface Methane Concentrations Along the Mid-Atlantic Bight Driven by Aerobic Subsurface Production Rather Than Seafloor Gas Seeps. *Journal of Geophysical Research: Oceans*, *125*(5), e2019JC015989. https://doi.org/10.1029/2019JC015989

Leonte, M., Wang, B., Socolofsky, S. A., Mau, S., Breier, J. A., & Kessler, J. D. (2018). Using Carbon Isotope Fractionation to Constrain the Extent of Methane Dissolution Into the Water Column Surrounding a Natural Hydrocarbon Gas Seep in the Northern Gulf of Mexico. *Geochemistry, Geophysics, Geosystems*, *19*(11), 4459–4475. https://doi.org/10.1029/2018GC007705

Li, L., Xue, B., Yao, S., Tao, Y., & Yan, R. (2018). Spatial–temporal patterns of methane dynamics in Lake Taihu. *Hydrobiologia*, *822*(1), 143–156. https://doi.org/10.1007/s10750-018-3670-4

Li, N., Jin, M., Peckmann, J., Chen, D., & Feng, D. (2023). Quantification of the sources of sedimentary organic carbon at methane seeps: A case study from the South China Sea. *Chemical Geology*, *627*, 121463. https://doi.org/10.1016/j.chemgeo.2023.121463

Li, Y., Xie, H., Scarratt, M., Damm, E., Bourgault, D., Galbraith, P. S., & Wallace, D. W. R. (2021). Dissolved methane in the water column of the Saguenay Fjord. *Marine Chemistry*, *230*, 103926. https://doi.org/10.1016/j.marchem.2021.103926

Li, Y., Xie, H., Scarratt, M. G., Damm, E., Galbraith, P. S., Lambert, N., Geng, L., & Wallace, D. W. R. (2022). Dissolved Methane in the World's Largest Semi-Enclosed Estuarine System: The Estuary and Gulf of St. Lawrence (Canada). *Journal of Geophysical Research: Oceans*, *127*(8), e2022JC018850. https://doi.org/10.1029/2022JC018850

Lindholm, M., Hessen, D. O., Mosepele, K., & Wolski, P. (2007). Food webs and energy fluxes on a seasonal floodplain: The influence of flood size. *Wetlands*, *27*(4), 775–784. https://doi.org/10.1672/0277-5212(2007)27[775:FWAEFO]2.0.CO;2

Lorenson, T. D., Greinert, J., & Coffin, R. B. (2016). Dissolved methane in the Beaufort Sea and the Arctic Ocean, 1992–2009; sources and atmospheric flux. *Limnology and Oceanography*, *61*(S1), S300–S323. https://doi.org/10.1002/lno.10457

Ma, X., Sun, M., Lennartz, S. T., & Bange, H. W. (2020). A decade of methane measurements at the Boknis Eck Time Series Station in Eckernförde Bay (southwestern Baltic Sea). *Biogeosciences*, *17*(13), 3427–3438. https://doi.org/10.5194/bg-17-3427-2020

Malakhova, T. V, Khurchak, A. I., Voitsekhovskaia, V. V, & Fedirko, A. V. (2024). Distribution of methane in the upper water layer of the northern Black Sea: Seasonal and daily trends and seawater-air emissions. *Continental Shelf Research*, *281*, 105320. https://doi.org/10.1016/j.csr.2024.105320

Mandryk, R. R., Capelle, D. W., Manning, C. C. M., Tortell, P., McCulloch, R. D., & Papakyriakou, T. (2021). First estimation of the diffusive methane flux and concentrations from Lake Winnipeg, a large, shallow and eutrophic lake. *Journal of Great Lakes Research*, *47*(3), 741–750. https://doi.org/10.1016/j.jglr.2021.03.011

Marinho, C., Palma-Silva, C., Albertoni, E., Trindade, C., & Esteves, F. A. (2009). Seasonal dynamics of methane in the water column of two subtropical lakes differing in trophic status. *Brazilian Journal of Biology = Revista Brasleira de Biologia*, *69*, 281–287. https://doi.org/10.1590/S1519-69842009000200007

Mastalerz, V., de Lange, G. J., Dählmann, A., & Feseker, T. (2007). Active venting at the Isis mud volcano, offshore Egypt: Origin and migration of hydrocarbons. *Chemical Geology*, *246*(1), 87–106. https://doi.org/10.1016/j.chemgeo.2007.09.005

Mendoza-Pascual, M. U., Itoh, M., Aguilar, J. I., Padilla, K. S. A. R., Papa, R. D. S., & Okuda, N. (2021). Controlling Factors of Methane in Tropical Lakes of Different Depths. *Journal of Geophysical Research: Biogeosciences*, *126*(4), e2020JG005828. https://doi.org/10.1029/2020JG005828

Middelburg, J. J., Nieuwenhuize, J., Iversen, N., Høgh, N., de Wilde, H., Helder, W., Seifert, R., & Christof, O. (2002). Methane distribution in European tidal estuaries. *Biogeochemistry*, *59*(1), 95–119. https://doi.org/10.1023/A:1015515130419

Morana, C., Borges, A. V, Roland, F. A. E., Darchambeau, F., Descy, J.-P., & Bouillon, S. (2015). Methanotrophy within the water column of a large meromictic tropical lake (Lake Kivu, East Africa). *Biogeosciences*, *12*(7), 2077–2088. https://doi.org/10.5194/bg-12-2077-2015

Morana, C., Bouillon, S., Nolla-Ardèvol, V., Roland, F. A. E., Okello, W., Descy, J.-P., Nankabirwa, A., Nabafu, E., Springael, D., & Borges, A. V. (2020). Methane paradox in tropical lakes? Sedimentary fluxes rather than pelagic production in oxic conditions sustain methanotrophy and emissions to the atmosphere. *Biogeosciences*, *17*(20), 5209–5221.

https://doi.org/10.5194/bg-17-5209-2020

Mu, C., Zhang, T., Wu, Q., Peng, X., Zhang, P., Yang, Y., Hou, Y., Zhang, X., & Cheng, G. (2016). Dissolved organic carbon, CO2, and CH4 concentrations and their stable isotope ratios in thermokarst lakes on the Qinghai-Tibetan Plateau. *J. Limnol*, *75*(2), 313–319.

Münchberger, W., Knorr, K.-H., Blodau, C., Pancotto, V. A., & Kleinebecker, T. (2019). Zero to moderate methane emissions in a densely rooted, pristine Patagonian bog – biogeochemical controls as revealed from isotopic evidence. *Biogeosciences*, *16*(2), 541–559. https://doi.org/10.5194/bg-16-541-2019

Murase, J., Sakai, Y., Sugimoto, A., Okubo, K., & Sakamoto, M. (2003). Sources of dissolved methane in Lake Biwa. *Limnology*, *4*(2), 91–99. https://doi.org/10.1007/s10201-003-0095-0

Natchimuthu, S., Panneer Selvam, B., & Bastviken, D. (2014). Influence of weather variables on methane and carbon dioxide flux from a shallow pond. *Biogeochemistry*, *119*(1), 403–413. https://doi.org/10.1007/s10533-014-9976-z

Orif, M., Kavil, Y., Kelassanthodi, R., Al-Farawati, R., & Alzubiadi, M. (2017). Dissolved methane and Oxygen depletion in the two coastal lagoons, Red Sea. *Indian Journal of Geo-Marine Sciences*, *46*.

Pain, A. J., Martin, J. B., Martin, E. E., Rennermalm, Å. K., & Rahman, S. (2021). Heterogeneous CO2 and CH4 content of glacial meltwater from the Greenland Ice Sheet and implications for subglacial carbon processes. *The Cryosphere*, *15*(3), 1627–1644. https://doi.org/10.5194/tc-15-1627-2021

Palma-Silva, C., Marinho, C. C., Albertoni, E. F., Giacomini, I. B., Figueiredo Barros, M. P., Furlanetto, L. M., Trindade, C. R. T., & Esteves, F. de A. (2013). Methane emissions in two small shallow neotropical lakes: The role of temperature and trophic level. *Atmospheric Environment*, *81*, 373–379. https://doi.org/10.1016/j.atmosenv.2013.09.029

Peacock, M., Audet, J., Jordan, S., Smeds, J., & Wallin, M. B. (2019). Greenhouse gas emissions from urban ponds are driven by nutrient status and hydrology. *Ecosphere*, *10*(3), e02643. https://doi.org/10.1002/ecs2.2643

Peacock, M., Davidson, S. J., Kothawala, D. N., Segersten, J., & Futter, M. N. (2023). Spatial and Seasonal Variations in Dissolved Methane Across a Large Lake. *Journal of Geophysical Research: Biogeosciences*, *128*(8), e2023JG007668. https://doi.org/10.1029/2023JG007668

Pickard, A., White, S., Bhattacharyya, S., Carvalho, L., Dobel, A., Drewer, J., Jamwal, P., & Helfter, C. (2021). Greenhouse gas budgets of severely polluted urban lakes in India. *Science of The Total Environment*, *798*, 149019. https://doi.org/10.1016/j.scitotenv.2021.149019

Pohlman, J. W., Knies, D. L., Grabowski, K. S., DeTurck, T. M., Treacy, D. J., & Coffin, R. B. (2000). Sample distillation/graphitization system for carbon pool analysis by accelerator mass spectrometry (AMS). *Nuclear Instruments and Methods in Physics Research Section B: Beam Interactions with Materials and Atoms*, *172*(1), 428–433. https://doi.org/10.1016/S0168-583X(00)00153-1

Quadra, G. R., Sobek, S., Paranaíba, J. R., Isidorova, A., Roland, F., do Vale, R., & Mendonça, R. (2020). High organic carbon burial but high potential for methane ebullition in the sediments of an Amazonian hydroelectric reservoir. *Biogeosciences*, *17*(6), 1495–1505. https://doi.org/10.5194/bg-17-1495-2020

Rehder, Z., Zaplavnova, A., & Kutzbach, L. (2021). Identifying Drivers Behind Spatial Variability of Methane Concentrations in East Siberian Ponds. *Frontiers in Earth Science*, *9*.

https://www.frontiersin.org/journals/earth-science/articles/10.3389/feart.2021.617662

Repo, M. E., Huttunen, J. T., Naumov, A. V, Chichulin, A. V, Lapshina, E. D., Bleuten, W., & Martikainen, P. J. (2007). Release of CO2 and CH4 from small wetland lakes in western Siberia. *Tellus B: Chemical and Physical Meteorology*. https://doi.org/10.1111/j.1600-0889.2007.00301.x

Riera, J. L., Schindler, J. E., & Kratz, T. K. (1999). Seasonal dynamics of carbon dioxide and methane in two clear-water lakes and two bog lakes in northern Wisconsin, U.S.A. *Canadian Journal of Fisheries and Aquatic Sciences*, *56*(2), 265–274. https://doi.org/10.1139/f98-182

Rosentreter, J. A., Borges, A. V, Deemer, B. R., Holgerson, M. A., Liu, S., Song, C., Melack, J., Raymond, P. A., Duarte, C. M., Allen, G. H., Olefeldt, D., Poulter, B., Battin, T. I., & Eyre, B. D. (2021). Half of global methane emissions come from highly variable aquatic ecosystem sources. *Nature Geoscience*, *14*(4), 225–230. https://doi.org/10.1038/s41561-021-00715-2

Rosentreter, J. A., Maher, D. T., Erler, D. V, Murray, R. H., & Eyre, B. D. (2018). Methane emissions partially offset "blue carbon" burial in mangroves. *Science Advances*, *4*(6), eaao4985. https://doi.org/10.1126/sciadv.aao4985

Sapper, S. E., Jørgensen, C. J., Schroll, M., Keppler, F., & Christiansen, J. R. (2023). Methane emissions from subglacial meltwater of three alpine glaciers in Yukon, Canada. *Arctic, Antarctic, and Alpine Research*, *55*(1), 2284456. https://doi.org/10.1080/15230430.2023.2284456

Sauer, S., Knies, J., Lepland, A., Chand, S., Eichinger, F., & Schubert, C. J. (2015). Hydrocarbon sources of cold seeps off the Vesterålen coast, northern Norway. *Chemical Geology*, *417*, 371–382. https://doi.org/10.1016/j.chemgeo.2015.10.025

Schmid, M., Batist, M. De, Granin, N. G., Kapitanov, V. A., McGinnis, D. F., Mizandrontsev, I. B., Obzhirov, A. I., & Wüest, A. (2007). Sources and sinks of methane in Lake Baikal: A synthesis of measurements and modeling. *Limnology and Oceanography*, *52*(5), 1824–1837. https://doi.org/10.4319/lo.2007.52.5.1824

Shakhova, N., Semiletov, I. P., & Bel'cheva, N. N. (2007). The great Siberian rivers as a source of methane on the Russian Arctic shelf. *Doklady Earth Sciences*, *415*(1), 734–736. https://doi.org/10.1134/S1028334X07050169

Shakhova, N., Semiletov, I., Salyuk, A., Yusupov, V., Kosmach, D., & Gustafsson, Ö. (2010). Extensive methane venting to the atmosphere from sediments of the East Siberian Arctic Shelf. *Science*, *327*(5970). https://doi.org/10.1126/science.1182221

Shirodkar, G., Naqvi, S. W. A., Naik, H., Pratihary, A. K., Kurian, S., & Shenoy, D. M. (2018). Methane dynamics in the shelf waters of the West coast of India during seasonal anoxia. *Marine Chemistry*, *203*, 55–63. https://doi.org/10.1016/j.marchem.2018.05.001

Shirokova, L. S., Pokrovsky, O. S., Kirpotin, S. N., Desmukh, C., Pokrovsky, B. G., Audry, S., & Viers, J. (2013). Biogeochemistry of organic carbon, CO2, CH4, and trace elements in thermokarst water bodies in discontinuous permafrost zones of Western Siberia. *Biogeochemistry*, *113*(1), 573–593. https://doi.org/10.1007/s10533-012-9790-4

Sieczko, A. K., Demeter, K., Singer, G. A., Tritthart, M., Preiner, S., Mayr, M., Meisterl, K., & Peduzzi, P. (2016). Aquatic methane dynamics in a human-impacted river-floodplain of the Danube. *Limnology and Oceanography*, *61*(S1), S175–S187. https://doi.org/10.1002/lno.10346

Solomon, E. A., Kastner, M., MacDonald, I. R., & Leifer, I. (2009). Considerable methane fluxes to the atmosphere from hydrocarbon seeps in the Gulf of Mexico. *Nature Geoscience*, *2*(8), 561–565. https://doi.org/10.1038/ngeo574

Sparrow, K. J., & Kessler, J. D. (2017). Efficient collection and preparation of methane from low concentration waters for natural abundance radiocarbon analysis. *Limnology and Oceanography: Methods*, *15*(7), 601–617. https://doi.org/10.1002/lom3.10184

Stanley, E. H., Loken, L. C., Casson, N. J., Oliver, S. K., Sponseller, R. A., Wallin, M. B., Zhang, L., & Rocher-Ros, G. (2023). GRiMeDB: the Global River Methane Database of concentrations and fluxes. *Earth Syst. Sci. Data*, *15*(7), 2879–2926. https://doi.org/10.5194/essd-15-2879-2023

Steinbach, J., Holmstrand, H., Shcherbakova, K., Kosmach, D., Brüchert, V., Shakhova, N., Salyuk, A., Sapart, C., Chernykh, D., Noormets, R., Semiletov, I., & Gustafsson, Ö. (2021). Source apportionment of methane escaping the subsea permafrost system in the outer Eurasian Arctic Shelf. *Proceedings of the National Academy of Sciences of the United States of America*, *118*(10), e2019672118. https://doi.org/10.1073/pnas.2019672118

Steinle, L., Graves, C. A., Treude, T., Ferré, B., Biastoch, A., Bussmann, I., Berndt, C., Krastel, S., James, R. H., Behrens, E., Böning, C. W., Greinert, J., Sapart, C.-J., Scheinert, M., Sommer, S., Lehmann, M. F., & Niemann, H. (2015). Water column methanotrophy controlled by a rapid oceanographic switch. *Nature Geoscience*, *8*(5), 378–382. https://doi.org/10.1038/ngeo2420

Taillardat, P., Linkhorst, A., Deblois, C. P., Prijac, A., Gandois, L., Tremblay, A., & Garneau, M. (2024). A Carbon Source in a Carbon Sink: Carbon Dioxide and Methane Dynamics in Open-Water Peatland Pools. *Global Biogeochemical Cycles*, *38*(4), e2023GB007909. https://doi.org/10.1029/2023GB007909

Teodoru, C. R., Nyoni, F. C., Borges, A. V, Darchambeau, F., Nyambe, I., & Bouillon, S. (2015). Dynamics of greenhouse gases (CO_2, CH_4, N_2O) along the Zambezi River and major tributaries, and their importance in the riverine carbon budget. *Biogeosciences*, *12*(8), 2431–2453. https://doi.org/10.5194/bg-12-2431-2015

Townsend-Small, A., Åkerström, F., Arp, C. D., & Hinkel, K. M. (2017). Spatial and Temporal Variation in Methane Concentrations, Fluxes, and Sources in Lakes in Arctic Alaska. *Journal of Geophysical Research: Biogeosciences*, *122*(11), 2966–2981. https://doi.org/10.1002/2017JG004002

Ueda, S., Go, C.-S. U., Yoshioka, T., Yoshida* ast;, N., Wada, E., Miyajima* ast ast;, T., Sugimoto, A., Boontanon, N., Vijarnsorn, P., & Boonprakub, S. (2000). Dynamics of dissolved O2, CO2, CH4, and N2O in a tropical coastal swamp in southern Thailand. *Biogeochemistry*, *49*(3), 191–215. https://doi.org/10.1023/A:1006393214432

Upstill-Goddard, R. C., Salter, M. E., Mann, P. J., Barnes, J., Poulsen, J., Dinga, B., Fiske, G. J., & Holmes, R. M. (2017). The riverine source of CH4 and N2O from the Republic of Congo, western Congo Basin. *Biogeosciences*, *14*(9), 2267–2281. https://doi.org/10.5194/bg-14-2267-2017

Wand, U., Samarkin, V. A., Nitzsche, H.-M., & Hubberten, H.-W. (2006). Biogeochemistry of methane in the permanently ice-covered Lake Untersee, central Dronning Maud Land, East Antarctica. *Limnology and Oceanography*, *51*(2), 1180–1194. https://doi.org/10.4319/lo.2006.51.2.1180

Wang, B., Stirling, E., He, Z., Ma, B., Zhang, H., Zheng, X., Xiao, F., & Yan, Q. (2021). Pollution alters methanogenic and methanotrophic communities and increases dissolved methane in small ponds. *Science of The Total Environment*, *801*, 149723. https://doi.org/10.1016/j.scitotenv.2021.149723

Wang, D., Chen, Z., Sun, W., Hu, B., & Xu, S. (2009). Methane and nitrous oxide concentration and emission flux of Yangtze Delta plain river net. *Science in China Series B: Chemistry*, *52*(5), 652–661. https://doi.org/10.1007/s11426-009-0024-0

Wang, H., Huang, R., Li, J., Chen, Q., & Ma, T. (2021). Dissolved and emitted methane in the Poyang Lake. *Science China Technological Sciences*, *64*(1), 203–212. https://doi.org/10.1007/s11431-020-1594-6

Wang, L., Xiao, C.-D., Du, Z.-H., Maher, D. T., Liu, J.-F., & Wei, Z.-Q. (2022). In-situ measurement on air–water flux of CH4, CO2 and their carbon stable isotope in lakes of northeast Tibetan Plateau. *Advances in Climate Change Research*, *13*(2), 279–289.

Wang, R., Zhang, H., Zhang, W., Zheng, X., Butterbach-Bahl, K., Li, S., & Han, S. (2020). An urban polluted river as a significant hotspot for water–atmosphere exchange of CH4 and N2O. *Environmental Pollution*, *264*, 114770. https://doi.org/10.1016/j.envpol.2020.114770

Wang, X., Yu, L., Liu, T., He, Y., Wu, S., Chen, H., Yuan, X., Wang, J., Li, X., Li, H., Que, Z., Qing, Z., & Zhou, T. (2022). Methane and nitrous oxide concentrations and fluxes from heavily polluted urban streams: Comprehensive influence of pollution and restoration. *Environmental Pollution*, *313*, 120098. https://doi.org/10.1016/j.envpol.2022.120098

Weber, T., Wiseman, N. A., & Kock, A. (2019). Global ocean methane emissions dominated by shallow coastal waters. *Nature Communications*, *10*(1), 4584. https://doi.org/10.1038/s41467-019-12541-7

Yan, F., Du, Z., Pu, T., Xu, Q., Wang, L., Ma, R., Zhang, C., Yu, Z., li, C., & Kang, S. (2023). Isotopic composition and emission characteristics of CO2 and CH4 in glacial lakes of the Tibetan Plateau. *Environmental Research Letters*, *18*. https://doi.org/10.1088/1748-9326/aceb7b

Yang, P., Lai, D. Y. F., Yang, H., Tong, C., Lebel, L., Huang, J., & Xu, J. (2019). Methane Dynamics of Aquaculture Shrimp Ponds in Two Subtropical Estuaries, Southeast China: Dissolved Concentration, Net Sediment Release, and Water Oxidation. *Journal of Geophysical Research: Biogeosciences*, *124*(6), 1430–1445. https://doi.org/10.1029/2018JG004794

Ye, W., Zhang, G., Zheng, W., Zhang, H., & Wu, Y. (2019). Methane distributions and sea-to-air fluxes in the Pearl River Estuary and the northern South China sea. *Deep Sea Research Part II: Topical Studies in Oceanography*, *167*, 34–45. https://doi.org/10.1016/j.dsr2.2019.06.016

Zhang, Y., Wang, X., Gong, X., Wu, S., Yuan, X., Liu, T., & Hou, C. (2021). Greenhouse gases concentrations and emissions in different inland water bodies in Chengdu Plain. *Desalination and Water Treatment*, *239*, 101–117. https://doi.org/10.5004/dwt.2021.27800

Zheng, Y., Wu, S., Xiao, S., Yu, K., Fang, X., Xia, L., Wang, J., Liu, S., Freeman, C., & Zou, J. (2022). Global methane and nitrous oxide emissions from inland waters and estuaries. *Global Change Biology*, *28*(15), 4713–4725. https://doi.org/10.1111/gcb.16233

Zhou, H., Yin, X., Yang, Q., Wang, H., Wu, Z., & Bao, S. (2009). Distribution, source and flux of methane in the western Pearl River Estuary and northern South China Sea. *Marine Chemistry*, *117*(1), 21–31. https://doi.org/10.1016/j.marchem.2009.07.011

Zigah, P. K., Oswald, K., Brand, A., Dinkel, C., Wehrli, B., & Schubert, C. J. (2015). Methane oxidation pathways and associated methanotrophic communities in the water column of a tropical lake. *Limnology and Oceanography*, *60*(2), 553–572. https://doi.org/10.1002/lno.10035
